# Bacterial extracellular vesicles control murine norovirus infection through modulation of antiviral immune responses

**DOI:** 10.3389/fimmu.2022.909949

**Published:** 2022-08-04

**Authors:** Sutonuka Bhar, Guanqi Zhao, Julia D. Bartel, Heather Sterchele, Alexa Del Mazo, Lisa E. Emerson, Mariola J. Edelmann, Melissa K. Jones

**Affiliations:** Department of Microbiology and Cell Science, Institute of Food and Agricultural Sciences (IFAS), University of Florida, Gainesville, FL, United States

**Keywords:** murine norovirus, commensal bacteria, outer membrane vesicles (OMVs), bacterial extracellular vesicles (bEV), microbiome, QuantiGene, antiviral immunity, innate immunity

## Abstract

Human norovirus is the primary cause of non-bacterial gastroenteritis globally and is the second leading cause of diarrheal deaths in children in developing countries. However, effective therapeutics which prevent or clear norovirus infection are not yet available due to a lack of understanding regarding norovirus pathogenesis. Evidence shows that noroviruses can bind to the surface of commensal bacteria, and the presence of these bacteria alters both acute and persistent murine norovirus infection through the modulation of host immune responses. Interestingly, norovirus-bacterial interactions also affect the bacteria by inducing bacterial stress responses and increasing the production of bacterial extracellular vesicles. Given the established ability of these vesicles to easily cross the intestinal barriers, enter the lamina propria, and modulate host responses, we hypothesized that bacterial extracellular vesicles influence murine norovirus infection through modulation of the antiviral immune response. In this study, we show that murine norovirus can attach to purified bacterial vesicles, facilitating co-inoculation of target cells with both virus and vesicle. Furthermore, we have found that when murine noroviruses and vesicles are used to co-inoculate macrophages, viral infection is reduced compared to virus infection alone. Specifically, co-inoculation with bacterial vesicles results in higher production and release of pro-inflammatory cytokines in response to viral infection. Ultimately, given that murine norovirus infection increases bacterial vesicle production *in vivo*, these data indicate that bacterial vesicles may serve as a mechanism by which murine norovirus infection is ultimately controlled and limited to a short-term disease.

## Introduction

Commensal bacteria maintain the homeostasis of the intestine by regulating the Th17/Treg balance. This role involves cell-cell communication between bacteria and host cells which is mainly performed by outer membrane vesicles (OMVs) in the case of Gram-negative bacteria. Gram-negative bacteria release 20-300 nm OMVs which contain proteins, metabolites, nucleic acids, and lipids similar to that of their parent bacteria (1). OMVs have been used in augmenting adaptive immunity against antigens by inducing a strong innate immune response (2, 3). In fact, OMVs can be more potent in driving cellular and humoral immunity than their heat-inactivated or live-attenuated parent bacteria (4, 5). OMVs possess inherent adjuvancy that can initiate maturation and activation of dendritic cells, releasing cytokines such as IL-12, IL-18, and IL-6 both *in vitro* and *in vivo*, and subsequently activating Th1 and Th17 cells (4, 6, 7). A recent study by Bae et al (8) investigated the adjuvant effect of OMVs against influenza infections *via* activating innate immunity. Administration of OMVs in mice recruited and activated macrophages providing protection against multiple strains of influenza virus. On subsequent immunization by those OMVs and influenza virus, local and systemic T cell responses and influenza-specific antibodies were found, indicating that OMVs play a role in procuring long-term adaptive immunity (3). Moreover, because of their inherent adjuvant properties and ability to drive cellular and humoral immune responses, OMVs are increasingly being developed as vaccines for bacterial and viral infections, including COVID-19 (5, 9–14).

Human noroviruses are the leading cause of viral gastroenteritis and are responsible for causing 685 million cases worldwide annually (15). Norovirus infection is characterized by a rapid onset of diarrhea associated with excretion of high concentrations of virus, with symptoms abating rapidly (within 24-48 hrs) (16). Human noroviruses have been shown to target a variety of cell types *in vitro*, including epithelial cells and various immune cell types (17–20). Some of these cell types have also been identified as norovirus targets *in vivo*, but only in immunocompromised patients, chronically infected with the virus (21). Unfortunately, the cellular tropism for human norovirus in immunocompetent patients during acute infection has not yet been determined. Furthermore, while human noroviruses can replicate in laboratory cultivated cells, a robust cell culture system for this virus remains elusive. As a result, murine norovirus (MNV) has been widely used as a surrogate for investigating norovirus pathogenesis. This model system has shed light on norovirus replication strategies, immunity and molecular mechanisms of pathogenesis in a natural host (19, 22–28).

Using the MNV model system, we recently demonstrated that murine norovirus, like human norovirus, effectively binds to a wide range of commensal bacteria (29). Furthermore, MNV infection significantly increases the release of bacterial extracellular vesicles (bEVs) by the intestinal gut flora (30). Interactions between commensal bacteria and both human and murine noroviruses *in vitro* also enhance bEV production by inducing bacterial stress responses (30). Others have shown that bacteria that produce a higher quantity of OMVs in the presence of environmental stressors such as antibiotics demonstrate improved survival (31–33), and hypervesiculation during antibiotic treatment is a common mechanism used by these microbes to ensure the survival of the parent bacteria (19, 34). Previous studies have shown that antibiotic treatment of mice decreases murine norovirus (MNV) infection (19, 34). However, antibiotic treatment might increase OMV production in the remaining bacterial populations before MNV inoculation, which would then result in the inhibition of viral replication.

The increased presence of bEVs during MNV infection provides a heightened opportunity for these vesicles to impact viral replication. Indeed, commensal bacteria are well established to alter infection for every enteric viral pathogen investigated to date [reviewed in (35–38)]. Therefore, based on the immune-modulatory abilities of bEVs, we hypothesized that an increase in bEV concentrations produced by Gram-negative bacteria such as *Enterobacter cloacae*, *Bacteroides thetaiotaomicron* and *Salmonella* Typhimurium during MNV infection would modulate the innate immune responses to the virus leading to control of viral replication. *E. cloacae* and *B. thetaiotaomicron* are commonly found within the intestinal microbiome of mammals (39), while *S.* Typhimurium is a pathogenic bacterium that infects the intestinal tract (40). Characterizing the mechanisms of antiviral immune response, immune evasion by a virus, and manipulation of host immune responses by commensal bacteria and their vesicles will give us a big picture of how intestinal immune cells respond to norovirus infection after norovirus has interacted with gut commensal bacteria in the intestinal mucosa. In this first-of-its-kind study, we optimized OMV-MNV co-inoculation in macrophages, quantified the resulting cytokine production, and identified the ability of OMVs from multiple bacterial species to control viral infection.

## Materials and methods

### Cell cultures

RAW264.7 and HEK 293T cell lines (ATCC) were grown in Dulbecco’s Modified Eagle Medium (DMEM; Corning) with 2 mM L-glutamine, 4.5 g/L glucose and sodium pyruvate; supplemented with 10% Fetal Bovine Serum (FBS; R&D systems), 100 U/mL penicillin, and 100 mg/mL streptomycin. The cell lines were cryopreserved in 50% freezing medium and 50% DMEM.

Bone marrow-derived macrophages (BMDMs) were obtained from euthanized, inbred wild type BALB/c mice (provided by Dr. Stephanie Karst, University of Florida). Leg bones (tibia and femur) were dissected from the mouse and sterilized in 70% ethanol for 1 min. The joint ends of the bones were cut to access the growth plate. The bones were flushed to remove the soft bone marrow, and the flushed solution was centrifuged at 600 x g for 5 mins at 4°C. The pellet was resuspended in 1 mL dPBS, and then 14 mL of additional dPBS was added. The solutions were rested at room temperature for 15 mins, then poured through a cell strainer, and centrifuged at 600 x g for 5 mins at 4°C. The cell pellet was resuspended with 3 mL complete RPMI media and cells were counted. Accordingly, 1.6x10^7^ cells were plated in T75 tissue culture treated flasks in complete RPMI with 4 mL M-CSF (2500X stock) per flask. Cells were incubated at 37°C with 5% CO_2_ and 7 mL of media with fresh M-CSF was replaced every 3 days.

### Murine norovirus production

Recombinant murine norovirus-1 (MNV-1) was generated using plasmid pSPMNV-1. CW3 (provided by Dr. Stephanie Karst, University of Florida) as previously described (24). The plasmid (5 μg) was transfected into HEK 293T cells. The supernatant from transfection (MOI=0.05) was used to infect RAW264.7 cells. At 36-48 hrs post-infection (hpi), the cells were checked for ~90% cytopathological effect (CPE). The supernatant containing viruses was harvested. Purified viral pellets were obtained by ultracentrifugation (Beckman Optima XE-90) of the supernatant through a 25% sucrose cushion. Viral pellets were resuspended in dPBS and titrated using TCID_50_ assay (24). Mock virus (referred to as mock in cell culture studies) were prepared in the same way using dPBS in place of plasmid during the transfection step. All virus stocks were aliquoted and stored at -80°C upon receipt and were thawed on ice for 1 hr prior to use.

### Median tissue culture infectious dose (TCID_50_) assay

A standard TCID_50_ assay was performed as described previously (24). Briefly, 3x10^4^ RAW264.7 cells per well were seeded in a 96 well plate and allowed to attach overnight. Following removal of the media, the cells were inoculated with serial dilutions of a given sample. The plates were incubated in 37°C, 5% CO_2_ for 7 days. Each well was then checked for CPE and TCID_50_/mL was calculated using Reed-Muench method.

### Bacterial strains and growth conditions

Bacterial isolates of *Enterobacter cloacae* (ATCC 13047), *Bacteroides thetaiotaomicron* (ATCC 29148, provided by Dr. Stephanie Karst, University of Florida), and *Salmonella* Typhimurium UK-1 (ATCC 68169, provided by Dr. Mariola Edelmann, University of Florida) were used for the study. *E. cloacae* and *S.* Typhimurium were cultured in Luria Bertani (LB) medium having 1% NaCl under aerobic conditions with constant shaking at 220 rpm at 37°C. *B. thetaiotaomicron* was cultured in conditioned Brain and Heart Infusion (BHI) medium supplemented with 0.001% hemin under anaerobic conditions using anaerobic chambers and anaerobic gas generation packs (Thermo Fisher) at 37°C. All strains were cryopreserved in 50% medium and 50% glycerol.

### Production, isolation and purification of outer membrane vesicles

OMVs were purified as described previously (30, 41). *E. cloacae*, *B. thetaiotaomicron*, and *S.* Typhimurium broth cultures (200 mL) were grown overnight (~ 18 hrs) in the presence of MNV or mock inoculum. The supernatant was collected after centrifugation at 2,300 x g for 20 mins at 4°C and then ultracentrifuged (Beckman Coulter Optima XE-90) at 25,000 x g for 20 mins at 4°C. The clarified supernatant was then filtered with 0.22 µm PES membrane filters and ultracentrifuged again at 150,000 x g for 2 hrs at 4°C to pellet OMVs. The OMV pellet was washed by resuspending in dPBS and ultracentrifuged at 150,000 x g for 2 hrs at 4°C. The purified OMV pellets were resuspended in 500 µl dPBS supplemented with protease inhibitor cocktail (Thermo Fisher) and stored at 4°C for use within 2 weeks. OMVs were checked for bacterial contamination by plating on agar plates.

### Attachment of MNV with purified OMVs

OMVs were purified from *E. cloacae* and quantified using Nanoparticles tracking analysis. OMVs were incubated with MNV (1:1) at 37°C for 1 hr. A virus only sample was used as control and treated similarly. The samples were ultracentrifuged at 150,000 x g for 2 hrs to pellet OMVs. The supernatant containing unbound virus was discarded and the OMV pellets were resuspended in RNA lysis buffer. Then RNA was isolated from those samples and MNV was quantified using RT-qPCR.

### Scanning electron microscopy

Purified *E. cloacae* OMVs were fixed in 2% paraformaldehyde in PBS and filtered through 1:10 poly-L-lysine (Sigma-Aldrich) treated 0.2mm GTTP isopore membrane filters (Merck Millipore). Then the filtrates were fixed again with Trump’s fixative (EMS) and washed with 0.1 M sodium cacodylate (pH=7.24). The samples were post-fixed with 2% OsO4, washed with water, and then dehydrated in 25-100% graded ethanol series with 25% increments. Dehydrated samples were critical point dried by Tousimis Autosamdri-815 (Tousimis) and mounted onto a 12 mm carbon conductive adhesive tab and aluminium stub (EMS) having sputter gold/palladium coating. The grids were imaged using Hitachi SU-5000 FE-SEM (Hitachi High Technologies) and visualized using EM Wizard software.

### Negative staining and transmission electron microscopy

Negative staining was done on purified OMVs from *B. thetaiotaomicron* cultures inoculated with PBS (mock) or MNV. OMVs were resuspended in 2% paraformaldehyde and mounted onto a 400-mesh carbon-coated Formvar nickel grid. The grid was glow discharged using PELCO easiGlow (Ted Pella) and floated onto a droplet of lysate for 5 mins. The filter paper was used to remove excess solution and samples were fixed again, followed by staining the grid by floating it on top of 10 mL 1% aqueous uranyl acetate stain for 30 s. Excess stain was removed by filter paper, and the grid was air-dried and examined using FEI Tecnai G2 Spirit Twin TEM (FEI Corp.). Images were obtained using Gatan ultraScan 2k x 2k camera and visualized in Digital Micrograph software (Gatan Inc.).

### Bacterial inactivation assay

Bacterial culture was grown overnight (18 hrs). After 18 hrs, the bacterial culture was pelleted and washed with 1X PBS twice and diluted to a final concentration of 10^4^ CFU/mL in PBS (unless otherwise specified). This bacterial solution was heat-inactivated by incubating for 40 mins at 65°C with gentle mixing every 10 mins (19). The inactivated bacteria were plated to confirm the loss of growth.

### Co-inoculation of cells with MNV and bacteria/OMVs

RAW264.7 cells and murine BMDMs were plated 5x10^5^ cells/mL in a 12 well plate (Corning) and grown for 24 hrs and 48 hrs before infections, respectively. The OMVs were diluted to 5, 1, and 0.1 μg/mL in PBS. The inactivated bacteria (MOI=0.01 corresponding 10^4^ CFU/mL) or OMV solutions were incubated with MNV (MOI=5) or PBS at 37°C for 1 hr with gentle rotation prior to infection. Plated cells were then inoculated with 500 μl of the solutions into each well. The plates were rocked gently to mix and incubated at 37°C with 5% CO_2_ for 1 hr. The inoculum was removed from each well, and wells were washed with dPBS twice. Complete media was added to the wells, and the cells were grown for 18 hpi at 37°C with 5% CO_2_. The supernatant was then collected for MNV detection *via* TCID_50_ assay and ELISA, and pellets were lysed for RNA isolation and QuantiGene assay.

### RNA isolation, reverse transcription, and quantitative PCR for MNV detection

Extraction of RNA from bacteria or OMVs incubated with MNV was done using the Quick RNA MiniPrep kit (Zymo Research), following manufacturer’s instructions. The cDNA was generated from the RNA using M-MLV RT (Promega). The reaction product was added to a mixture of forward and reversed MNV primers (19) (**Supplementary Table 1**) and PowerUp SYBR Green Master Mix (Applied Biosystems), and real-time quantitative PCR was carried out in QuantiStudio3 (Thermo Fisher). Samples were heated for 10 mins at 95°C and amplified for 40 cycles of 15 s at 95°C, 60 s at 58°C, and 15 s at 75°C. Each run was completed with a melting curve to confirm amplification specificity and lack of primer dimers. The values were converted to MNV genome copies per sample using a standard curve obtained with each plate. The standard curve was generated using linearized pSPMNV-1.CW3 plasmid.

### RT-qPCR for ISG gene expression analysis

RNA was isolated from pellets obtained from co-inoculation of RAW264.7 and BMDM. The RNA concentration was measured using Nanodrop, and 2.5 μg RNA was taken for DNase treatment. The RNA was treated with 3U of DNase from the Turbo DNA-*free™* kit (Thermo Fisher) for 30 mins at 37°C. The reaction was stopped using the Inactivation reagent, and the clarified RNA was separated. Using 2 μg of DNA-free RNA, we performed reverse transcription (RT) using M-MLV RT (Promega) to obtain cDNA and negative control (1 μg each). The cDNA was adjusted in volume with nuclease-free water to have a concentration of 20 ng/reaction in qPCR. qPCR was performed to quantify the amount of IRF1, ISG15, MX1, MX2, IFIT1 cDNA in our samples. The list of primers in **Supplementary Table 1**. Each qPCR was performed in a 20 μL reaction volume (6 μl nuclease-free water, 10 μL 2× SYBR Green master mix (Thermo Fisher), 1 μL forward oligonucleotide [10 μM], 1 μL reverse oligonucleotide [10 μM], and 2 μL cDNA template [20 ng]). The qPCR was done in QuantStudio3 with 40 cycles of both 95°C for 15 secs followed by 60°C for 1 min. The PCR stage was followed by a Melt Curve Stage for process validation.

### Multiplex mRNA expression analysis

Multiplexed cytokine mRNA expression was quantified using QuantiGene™ Plex Gene Expression Assay following the manufacturer’s user guide. Briefly, RNA was purified from the macrophages and diluted to 1 μg/well. RNA was added in duplicates into a hybridization plate containing working beads mix (**Supplementary Tables 2, 3**) and incubated overnight in VorTemp™ 56 at 54°C. Then, the mixtures are transferred to the magnetic separation plate and washed to remove anything not attached to the beads. Pre-amplifier solution, amplifier solution, label probe, SAPE working reagent are hybridized to the magnetic beads, respectively. The plate is then washed, sealed, and read on MagPix™ instrument. The output data was normalized with HPRT1 mRNA readings for each sample and then again normalized with the mock readings for each cytokine mRNA transcript.

### Cell viability measurement by MTS assay

Macrophages were seeded in a 96-well plate at a concentration of 2x10^4^ cells/well and incubated in 37°C with 5% CO_2_ for 24 hrs (RAW264.7) or 48 hrs (BMDM). Wells were co-inoculated or treated with controls in duplicates and then washed, and cells were grown for 18 hpi. MTS reagent (Abcam) was added onto media and incubated for 2 hrs at 37°C with 5% CO_2_. The plate was shaken to mix the contents and absorbance was measured in Epoch microplate reader (BioTek) at OD=490 nm.

### Cell cytotoxicity quantification by LDH assay

Cells were seeded in a 96-well plate at 2x10^4^ cell/well and incubated in 37°C with 5% CO_2_ for 24 hrs (RAW264.7) or 48 hrs (BMDM). Wells were co-inoculated with OMVs + MNV or treated with OMVs or MNV alone. All treatments were performed in duplicate and then washed and grown for 18 hpi. CyQUANT™ LDH Cytotoxicity Assay (Invitrogen) kit was used to measure the LDH release from each well using manufacturer’s instructions. In brief, 50 μL of supernatants were transferred into a new plated and 50 μl of reaction mixture was added to each sample. Plate was gently tapped to mix and then kept in the dark at room temperature for 30 mins. 50 μl of stop solution was then added to each well and mixed and absorbance was measured immediately at 490 nm and 680 nm. The background (680 nm value) was subtracted and % cytotoxicity was measured using the following formula:


$$\%\;cytotoxicity=\frac{\left(Sample\;LDH\;activity-spontaneous\;LDH\;activity\right)}{\left(Maximum\;LDH\;activity-Spontaneous\;LDH\;activity\right)}\times 100$$

### OMV staining and fluorescent microscopy

Cells were seeded at a concentration of 2x10^4^ cells/well in a 96-well plate (Greiner) at least 12 hrs before treatment. Purified OMVs (0.2 mg) were stained using 0.1% Vybrant™ DiO fluorescent dye (Thermo Fisher) as described previously (42). Briefly, the OMV solution with dye was incubated at 37°C for 30 mins in the dark. Unbound dye was removed by centrifuging using 10kDA ultracentrifugal filters and washing with dPBS three times. The stained OMVs were diluted to the required dilutions in complete media before the experiment. Media was removed from the 96-well plate, and 40 μl of stained OMVs in complete media were inoculated. The plate was incubated for 1 hr at 37°C, 5% CO_2_. The inoculum was aspirated, and wells were washed with dPBS. Complete media was added to the wells, and cells were grown for the required amount of time before fixation. For cell fixing, the media was removed, and wells were washed twice with 1x HBSS and fixed with 4% PFA for 30 min. The cells were incubated with 1:1,000 DAPI for 7 mins at room temperature in the dark to stain the cell nucleus. DAPI was removed, and cells were washed again with 1x HBSS. The fixed cells can be stored in the dark at 4°C with 100 µL HBSS. Fluorescent readings were performed using Cytation 5 (Biotek, Agilent) with Gen5 (3.11) imaging software, and DiO-labeled OMVs were observed using a GFP filter (469/525nm).

### Enzyme linked immunosorbent assay (ELISA)

ELISAs were carried out using cell culture supernatants following the manufacturer’s instructions (R&D systems) for TNFα, IL-10, IFNβ, and IFNγ and Thermo Fisher for IFNα. Briefly, 96-well plates were coated overnight and blocked. Standards or samples containing cell culture supernatants were incubated for 1 hr. Detection was done by primary antibodies, HRP-conjugated secondary antibody, and substrate. Colorimetric reading was measured using a spectrophotometer (Bio-Rad). Data were analyzed using GraphPad Prism, and differences in cytokine expression to MNV samples were determined.

### Graphical and statistical analysis

All the graphs were designed in GraphPad Prism v9.3. Ordinary one-way ANOVA was used to analyze co-inoculation LDH cytotoxicity, QuantiGene cytokine expression, and TCID_50_ MNV output titer results. T-test with Welsch’s correction was done to detect significant changes in MNV attachment to live vs. inactivated bacteria, OMV attachment assay, and for validating co-purification using TCID_50_ assay.

## Results

### Attachment of murine noroviruses to inactivated bacteria and OMVs

**Figure 1:**
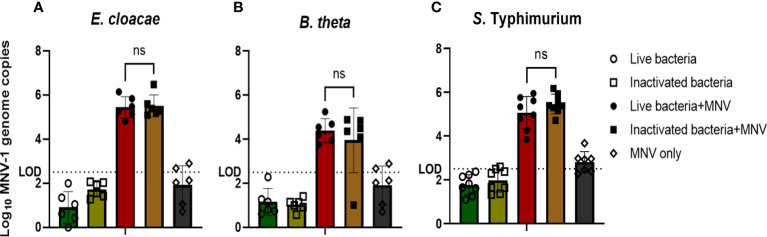
MNV binds to live and inactived bacteria at similar levels. **(A)** *E cloacae*, **(B)** *B thetaiotaomicron*, and **(C)** *S.* Typhimurium were incubated at room temperature (live) or at 65°C (heat inactivated) for 40 mins and subsequently incubated with MNV for 1 hr. After 2 washes of the bacterial pellet, MNV was quantified by RT-qPCR (n≥6). T-test with Welsch’s correction was done to detect significant changes in MNV attachment to live vs. inactivated bacteria. (ns= not significant).

Since, under the conditions used, live bacteria otherwise kill or activate macrophages used in MNV infection, the bacteria included in the co-inoculations needed to be inactivated, as has been demonstrated for *in vitro* infection of HNoV (19). However, the binding of murine noroviruses to heat-inactivated bacteria has never been examined. Therefore, we tested if heat inactivation of *E. cloacae*, *B. thetaiotaomicron*, and *S.* Typhimurium disrupted the binding of MNV to the bacterial cells. We found that similar amounts of MNV bound to inactivated bacteria as to live bacteria (**Figure 1**). An MNV-only control was used to ensure that the detected MNV was not a residual virus pelleted during washing or bound to the experimental tubes. Based on these results, heat-inactivated bacteria were used in later experiments.

**Figure 2:**
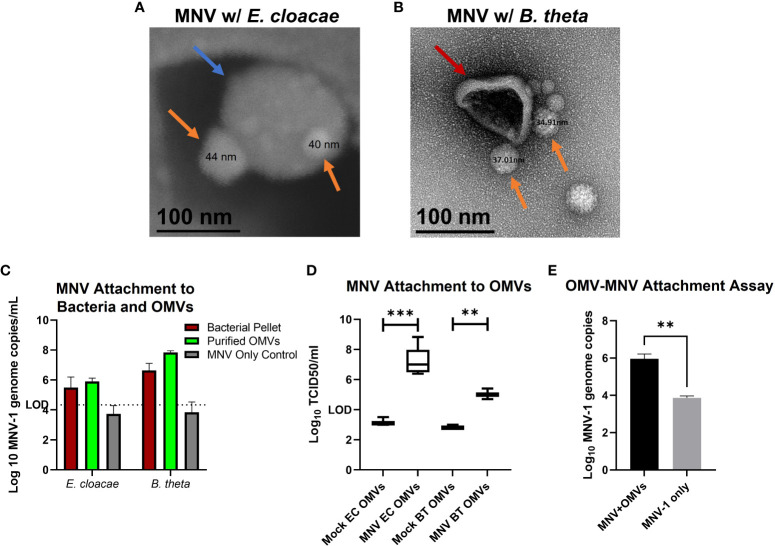
MNV attachment to OMVs. After incubation of bacterial culture with MNV, OMVs were collected by ultracentrifugation and MNV attachment to OMVs was assessed. **(A)** OMVs harvested from *E cloacae* cultures (blue arrow) were analyzed with scanning electron microscopy (SEM) to observe attached MNV (orange arrows). **(B)** OMVs derived from *B thetaiotaomicron* cultures (red arrows) were analyzed with transmission electron microscopy (TEM) to observe MNV (orange arrows) binding to OMVs, **(C)** RT-qPCR and **(D)** TCID_50_ assay were performed to quantify the co-purification of MNV with OMVs harvested from *E cloacae (*EC*)* and *B thetaiotaomicron* (BT) cultures incubated with MNV, **(E)** OMVs were harvested from naïve bacterial cultures and subsequently incubated with MNV. RT-qPCR analysis was performed to quantify MNV binding to free-floating OMVs in the absence of the parental bacteria. **=p<0.01, ***=p<0.001.

We have previously shown that MNV binds to OMVs as they bud from the surface of *E. cloacae* (30). We then questioned if the virus remained bound to OMVs after the budding process when the vesicles are released into the medium and move away from the parental bacterium. We performed SEM of OMVs harvested from *E. cloacae* cultures that were grown in the presence of MNV and found MNV (~40 nm in diameter as measured) attached on the surface of the OMVs. These images suggest that MNV can remain attached to OMVs even after vesicle purification (**Figure 2A**). Similarly, TEM imaging of OMVs harvested from *B. thetaiotaomicron* cultures grown in the presence of MNV also shows that the virus remains bound to the surface of vesicles produced by this bacterium (**Figure 2B**). Mock OMVs derived from the bacterial cultures in absence of MNV were imaged in parallel and did not show virus sized particles (data not shown). Next, we used RT-qPCR to quantify the amount of MNV bound to OMVs purified from *E. cloacae* and *B. thetaiotaomicron* that had been incubated with or without the virus. For these experiments, two controls were used: 1. the bacterial pellet was analyzed to ensure that our bacterial attachment assay was successful and that MNV bound to both bacterial strains, and 2. an MNV-only control was incubated, processed, and analyzed in parallel to ensure that any detected MNV in the OMV preparations was not due to residual viruses pelleting during ultracentrifugation or binding to the experimental tubes. The results showed MNV genome copies present in the OMV preparations, further confirming that MNV was successfully attached to *E. cloacae* and *B. thetaiotaomicron* OMVs as well as to the parental bacteria (**Figure 2C**). The MNV-only control did not contain any detectable virus, indicating that any unattached virus was removed during the wash steps. TCID_50_ analysis of these samples confirmed the RT-qPCR data, where MNV was detected in the OMVs derived from virus-exposed bacterial cultures but not from mock exposed cultures (**Figure 2D**). We also wanted to determine if MNV was capable of binding to free-floating OMVs in the absence of the parental bacterium. OMVs were isolated from *E. cloacae*, and the purified vesicles were incubated with MNV. After incubation, the vesicles were ultracentrifuged and washed to remove any unbound virus, and then viral titers were quantified using qRT-PCR. Results showed that MNV co-purified with the OMVs (**Figure 2E**). Since ultracentrifugation can also pellet viruses, a virus-only control (without OMVs) was also tested in parallel. These results showed that some free viruses were pelleted during ultracentrifugation, but the amount of free virus was significantly less than the amount detected in OMV+MNV samples (**Figure 2E**). Together, these results demonstrate that MNV is capable of binding to OMVs as they are being generated by their parental bacteria, but also after they have been produced and migrated away from the bacterial cell, potentially increasing their ability to interact with MNV within the host.

### Analysis of OMV uptake into macrophages

**Figure 3:**
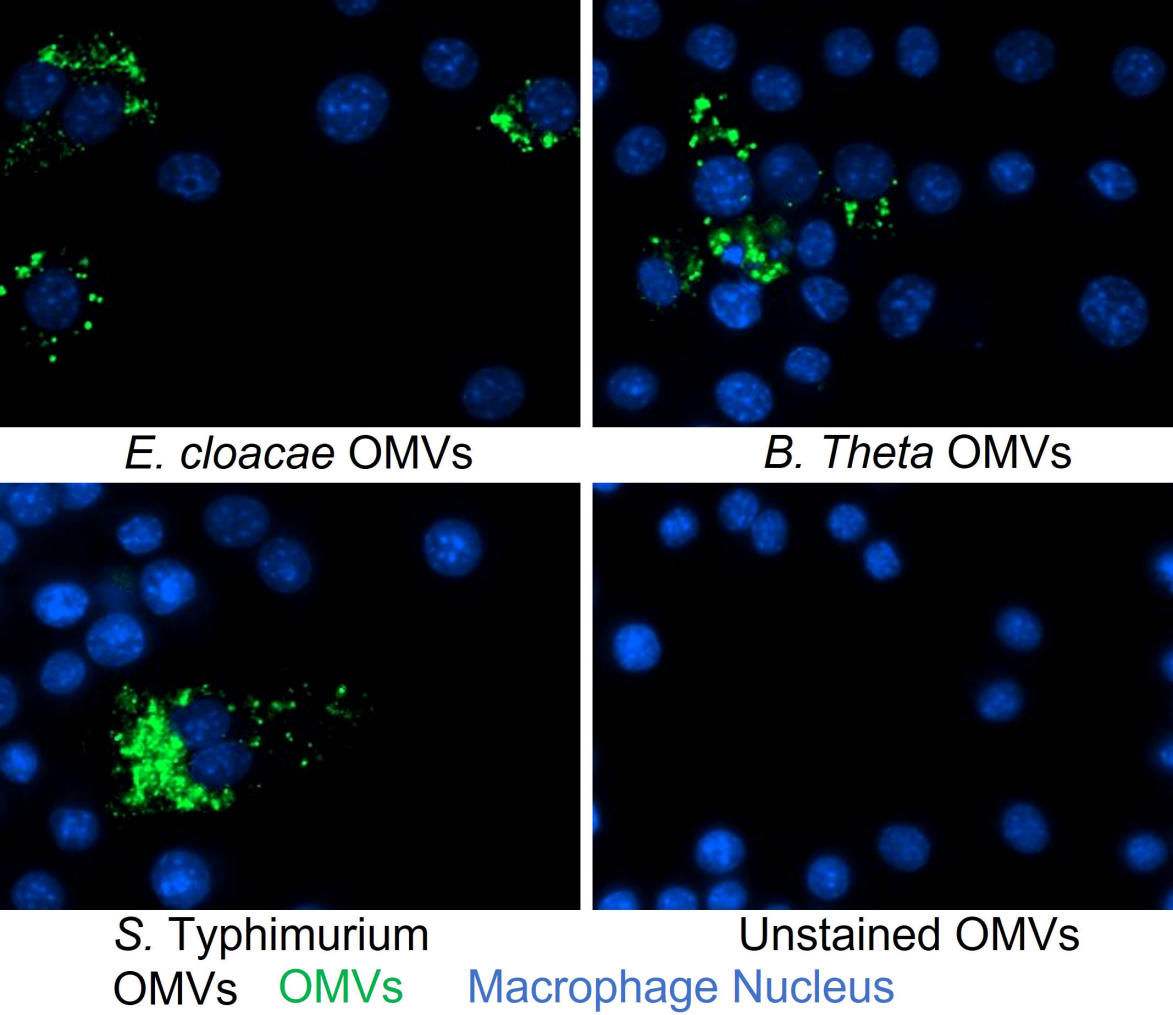
Analysis of OMVs uptake into macrophages at 1 hr post-inoculation. OMVs derived from *E cloacae*, *B thetaiotaomicron*, or *S.* Typhimurium were stained with DiO, inoculated onto RAW264.7 cells, and incubated at 37°C for 1 hr. The cells were washed and stained with DAPI during fixation and then visualized using the Cytation 5 multiplate reader equipped with a fluorescent microscope at a 20x magnification. Untreated control showed only DAPI staining of the nucleus (blue), whereas the OMV treated wells showed the presence of OMVs (green) surrounding the nucleus, suggesting the presence of OMVs in the macrophages.

Based on previous work demonstrating that OMVs from other commensal bacteria can enter cells and subsequently influence cellular responses (43–45), we examined the ability of OMVs from *E. cloacae* and *B. thetaiotaomicron* to enter RAW264.7 cells. In addition, since MNV has been shown to infect macrophages as early as 1 hpi, which is the standard incubation timepoint for MNV infection assays (26, 28), we tested if OMVs were capable of entering macrophages within the same time frame. To do this, RAW264.7 macrophages were inoculated with DiO-labeled OMVs, and the cells were visualized after 1 hr of inoculation with fluorescent microscopy. DiO-stained OMVs were visible as green, punctate staining surrounding the nucleus (blue), indicating that the vesicles had entered these cells within 1 hr time frame (**Figure 3**). These experiments demonstrate that *E. cloacae* and *B. thetaiotaomicron* OMVs can likely enter into the macrophages and also enter cells within a short time (1 hr), which should be verified by using more advanced microscopy techniques.

### Cell viability of macrophages after treatment with commensal bacteria and OMV-MNV co-inoculation

To optimize the CFU of inactivated bacteria to be tested in viral infection assays, various concentrations of bacteria (10^6^ CFU/mL, 10^4^ CFU/mL, and 10^2^ CFU/mL) were used to inoculate RAW264.7 macrophages and then MTS assay was performed to determine their impact on cell viability. For *E. cloacae* or *B. thetaiotaomicron* 10^6^ CFU/mL significantly decreased RAW264.7 cell viability (**Supplementary Figure 1**). Therefore, 10^4^ CFU/mL of each bacterium was chosen for further experiments. Next, the viability of RAW264.7 cells and BMDMs was determined after treatment with *E. cloacae* or *B. thetaiotaomicron* OMVs in the presence or absence of MNV. During initial experiments, cell viability was assessed using trypan blue, but no significant differences in cell viability were observed (data not shown). This result was unexpected given the cytotoxic nature of MNV infection on macrophages. Therefore, we performed MTS and LDH assays to measure cell viability and cytotoxicity, respectively.

**Figure 4:**
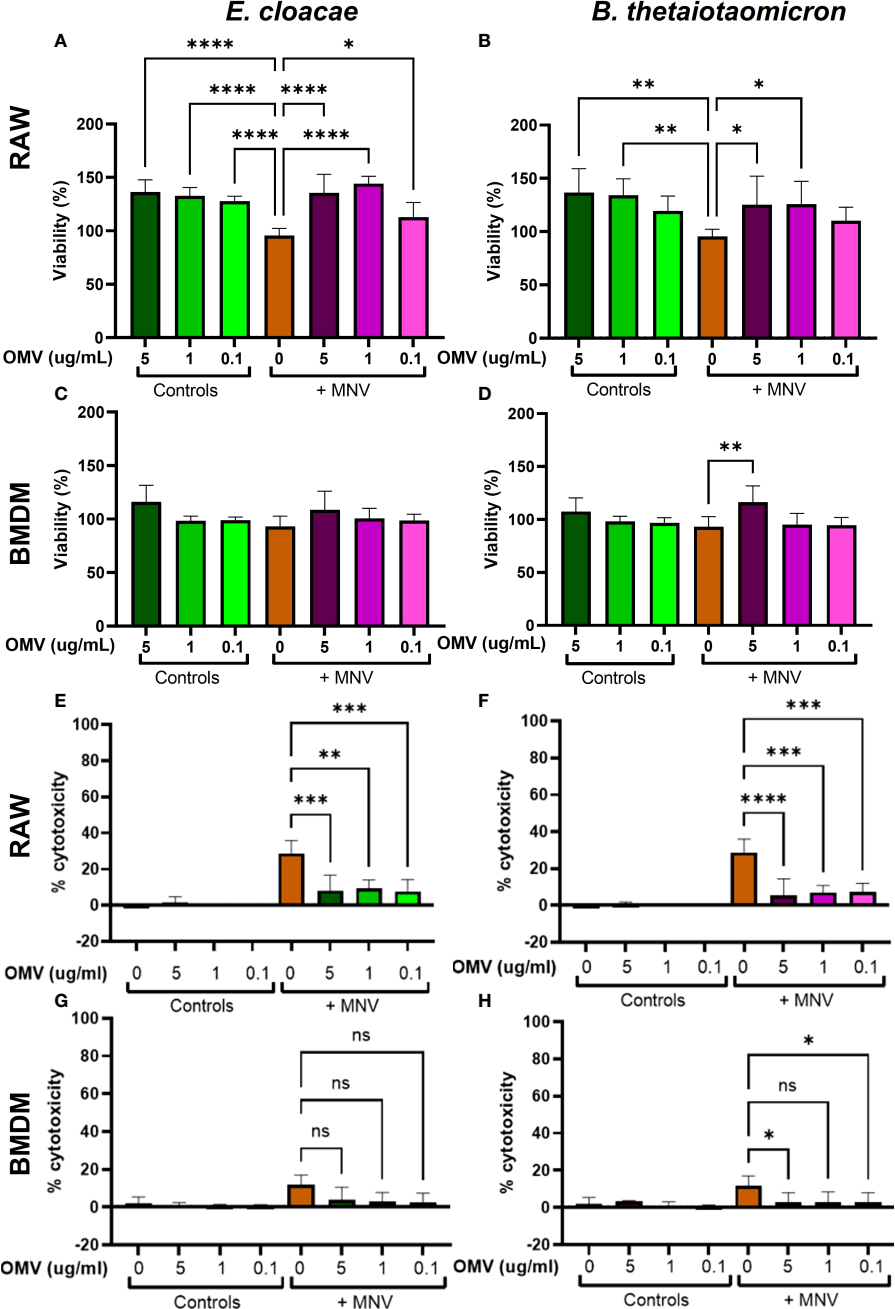
Viability and cytotoxicity of macrophages after infection with OMVs and MNV. RAW264.7 and BMDM cells were treated with mock virus, OMVs only, MNV only, or MNV+OMV. Cell viability of RAW264.7 cells **(A, B)** and BMDMs **(C, D)** was measured 18 hours post-infection with using MTS assay. Cell cytotoxicity as measured by LDH release was also measured at 18 hours post-treatment for RAW264.7 cells **(E, F)** and BMDMs **(G, H)** using spectrophotometry. One-way ANOVA was used to determine statistical significance. (n=3 for all experiments; *=p<0.05, **=p<0.01, ***=p<0.001, ****=p<0.0001). Ns= not significant.

The viability of macrophages treated with OMVs, MNV, OMV + MNV and mock inoculum were also measured and percent viability was determined as compared to the mock treated samples. Results showed that none of the OMV concentrations reduced the viability of either cell line, and hence, all three concentrations were used in further experiments (**Figures 4A–D**). Interestingly, while concentrations of OMVs as high as 5 μg/mL from both *E. cloacae and B. thetiotaomicron* did not significantly reduce macrophage viability, OMVs treatment often resulted in significant increases in percent viability as measured by MTT assay in both the presence and absence of virus, particularly in RAW264.7 cells (**Figures 4A, B**). This observation is likely due to OMV specific stimulation of the cell. This stimulation may indeed be increasing cell viability, but the observed increase may also simply be an artifact of the assay. The vesicles may be interfering with energy metabolism which would increase MTT and thus percent viability. Further experimentation will be required to directly investigate this observation. *S.* Typhimurium impacted cell viability in a similar manner compared to *E. cloacae* and *B. thetaiotaomicron* in RAW264.7 cells (**Supplementary Figure 2A**) but was not examined in BMDMs due to a lack of macrophages.

Next, the cytotoxicity of OMVs and MNV on RAW264.7 cells and BMDMs were measured by using the LDH assay. Cells were treated with the OMVs, OMVs and MNV, or with MNV alone. Results showed that cells exposed to MNV exhibited cytotoxicity after 18 hpi, and cytotoxicity was more pronounced in RAW264.7 cells than in BMDMs (**Figures 4E–H**). However, co-inoculation of OMVs and MNV significantly lowered virus induced cytotoxicity in RAW264.7 cells for both bacterial OMVs and at all OMV concentrations (**Figures 4E, F**). Interestingly, for BMDMs, while reductions in cytotoxicity were also observed in the presence of OMVs, only 5 µg and 0.1 µg of *B. thetaiotaomicron* OMVs yielded significantly less cytotoxicity and significant reductions were not observed for any *E. cloacae* OMV concentrations (**Figure 4H**). However, the lack of significant decreases in cytotoxicity in BMDMs may be due the low cytotoxic effect of MNV in BMDMs to begin with (**Figures 4G, H**). Incubation with *S*. Typhimurium resulted in similar levels of cytotoxicity compared to *E. cloacae* and *B. thetaiotaomicron* in RAW264.7 cells (**Supplementary Figure 2B**). The effect in BMDMs was not examined due to a shortage of macrophages. Overall, these results demonstrate that OMVs do not induce adverse effects on the macrophage cell types tested and may be beneficial to the cell as high concentrations of OMVs increased cell viability and reduced virus-related cytotoxicity.

### Changes in cytokine gene expression and production after co-inoculation

**Figure 5:**
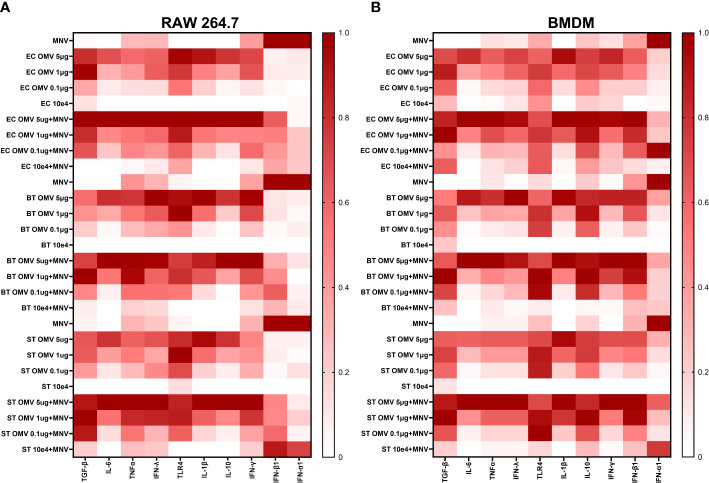
OMVs alter expression of antiviral and inflammatory cytokine genes during MNV infection of macrophages. RAW264.7 cells and BMDMs were treated with inactivated bacteria, OMVs only, MNV only, MNV+OMV, inactivated bacteria+MNV, or mock virus. Cells were harvested at 18 hours post infection and the RNA transcripts measured using the QuantiGene assay. Output data was normalized with HPRT1 mRNA readings for each sample and then normalized to the mock virus readings for each cytokine mRNA transcript. Heatmaps summarizing the change in transcript expression relative to mock for TGF-β, IL-6, TNFα, IFN-Λ, TLR4, IL-1β, IL-10, IFN-γ, IFN-β1, IFN-α1 transcripts. **(A)** RAW264.7 and **(B)** BMDMs. MNV-only infections were performed and analyzed separately for each bacterial OMV treatment.

**Figure 6:**
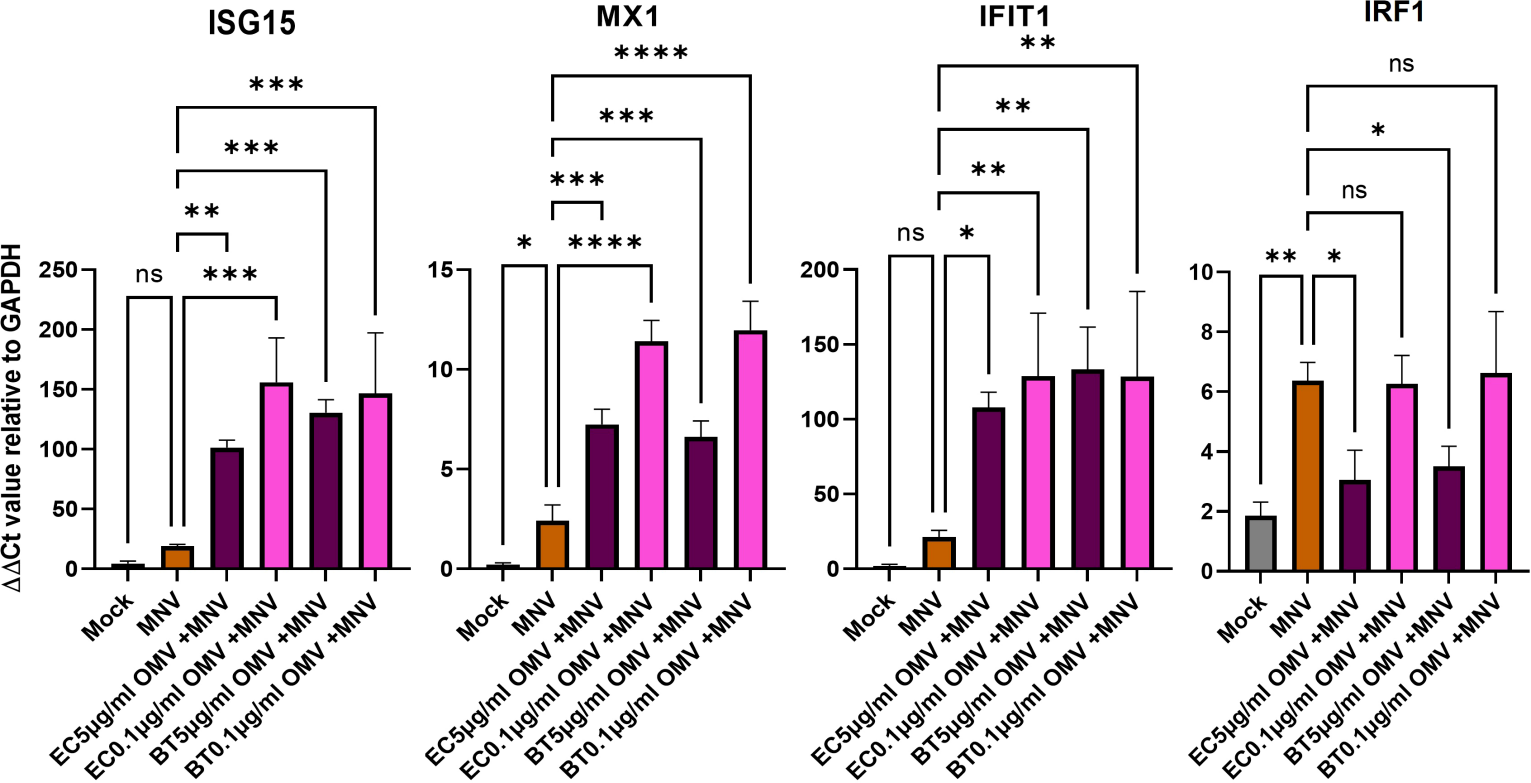
ISG gene expression analysis. RAW264.7 cells were infected with mock virus, MNV, OMVs, and OMVs+ MNV. ISG15, MX1, IFIT1, and IRF1 expression were measured in the cells at 18 hpi. All the treatments were compared to ISGs expression in MNV only infected RAW264.7. Ordinary one-way ANOVA was used to analyze qRT-PCR results. (n=3; ns= not significant, *=p<0.05, **=p<0.01, ***=p<0.001, ****=p<0.0001). Results showed that the presence of OMVs frequently induced significant changes in ISG expression. Ns= not significant.

Based on the established ability of OMVs produced by commensal bacteria to modulate innate immune responses (2, 3), their increased presence during MNV infection (30), and the ability of bacterial by-products to alter innate immune responses to MNV infection (21), we hypothesized these vesicles would alter immune responses during viral infection. To assess OMV-induced changes in the innate immune response during MNV infection, RAW264.7 or BMDMs cells were co-inoculated with MNV and inactivated bacteria (10^4^ CFU/mL) or OMVs (5 μg/mL, 1 μg/mL, and 0.1 μg/mL), followed by the measurement of transcripts for cytokines that are critical for control of MNV infection (23, 46–49) as well as other genes involved in immune response signaling influenced by commensal bacterial OMVs (43, 50, 51). MNV infection alone produced a low amount of TGF-β, IL-6, TNFα, IFN-Λ, TLR4, IL-1β, IL-10, and IFNγ transcripts for both the cell lines (**Figure 5**, **Supplementary Figure 3**). As the concentration of OMVs in the co-inoculated increased, the expression of these transcripts also increased. Interestingly, the same trend of mRNA expression increase was also observed for OMV only treatments. For RAW264.7, IFN-α and IFN-β were highly expressed in MNV infection, and expression of these transcripts was decreased as the OMV concentration increased during co-inoculation regardless of the bacterium from which the OMVs were derived (**Figure 5A**). IFN-α expression was similar in BMDMs, but the IFN- β expression was low for MNV only infection and increased as the OMV concentration increased in the treatments (**Figure 5B**). Notably, OMV treatment alone did not induce type I IFN production in RAW264.7 cells (**Figure 5A**) or IFN-α production in BMDMs (**Figure 5B**). Interestingly, cell treatments using inactivated bacteria only (*E. cloacae* and *B. thetaiotaomicron*) or inactivated bacteria + MNV resulted in small amounts of all the tested transcripts for both the cell lines (**Figure 5**). Since Type 1 IFNs induce expression of ISGs which are involved in upregulating antiviral responses, we also measured ISG expression using RT-qPCR. Results showed that ISG15, MX1 and IFIT1 could also be induced in the presence of OMVS, even at concentrations as low as 0.1 µg/mL (**Figure 6**). Furthermore, since IRF1 plays a significant role in the upregulation and activation of type I IFNs, we also measured IRF1 gene expression using RT-qPCR. In RAW264.7 cells, IRF1 expression decreased after co-inoculation of OMV+MNV compared to infection with MNV alone for both types of OMVs as well as both OMV concentrations tested (**Figure 6**). This result is consistent with the decrease in type I IFNs in RAW264.7 cells when 5 µg/mL of OMVs are present during MNV infection (**Figure 5**).

**Figure 7:**
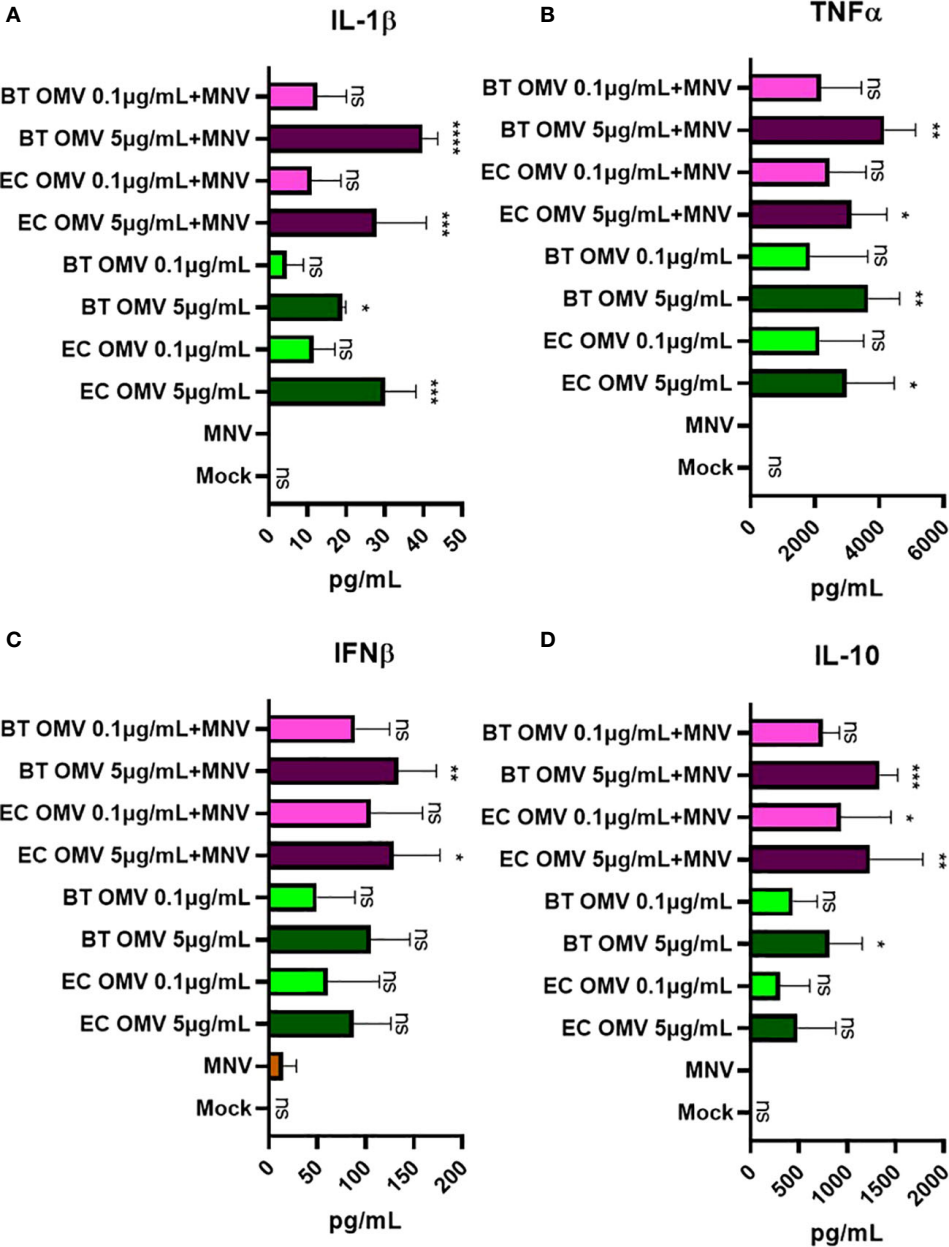
Alteration of the cytokine level secreted by macrophages on MNV-OMV co-inoculated. ELISA was used to measure the quantity of secreted cytokines in BMDM supernatant (n=3) obtained after treatments with mock, MNV, *E cloacae* OMVs, *B thetaiotaomicron* OMVs, MNV+*E. cloacae* OMVs, or MNV+ *B thetaiotaomicron* OMVs. A high concentration (5 μg/mL) and a low concentration (0.1 μg/mL) of OMVs were used. The following cytokines were measured **(A)** IL-1β, **(B)** TNFα, **(C)** IFNβ, and **(D)** IL-10, and statistical significance was calculated relative to MNV only infection supernatants. T-test with Welsch’s correction was done to detect significant changes in protein secretion. (*=p<0.05, **=p<0.01, ***=p<0.001, ****=p<0.0001). Ns= not significant.

To determine if gene expression correlated with changes in protein production, we quantified key cytokines in the culture supernatant. Consistent with gene expression data, ELISAs showed increased production of IL-1β, TNFα, IFNβ, and IL-10 in BMDMs co-inoculation with OMVs + MNV compared to MNV alone (**Figure 7**). While significant differences were generally only observed with high concentrations of OMVs (5 µg/mL), lower OMV concentrations still tended to result in higher protein levels than MNV alone. For the proteins examined, both gene expression (**Figure 5**) and ELISA data (**Figure 7**) indicate that OMVs from *E. cloacae* and *B. thetaiotaomicron* induce expression of specific antiviral immune pathways (IL-1β, TNFα, IFNβ) in the presence and absence of MNV, which is supported by upregulation of gene expression for additional cytokines and chemokines (IL-6, IFN-γ, IFN-ʎ, ISG15, MX1 and IFIT1; **Figures 5**, **6**). However, some inconsistencies with induction of antiviral pathways were also observed. Specifically, IL-10, which is not an antiviral modulator, was also upregulated in the presence of OMVs (**Figures 5**, **7**), demonstrating the need for further investigation to elucidate the immune pathways and downstream effects of OMVs during MNV infection.

**Figure 8:**
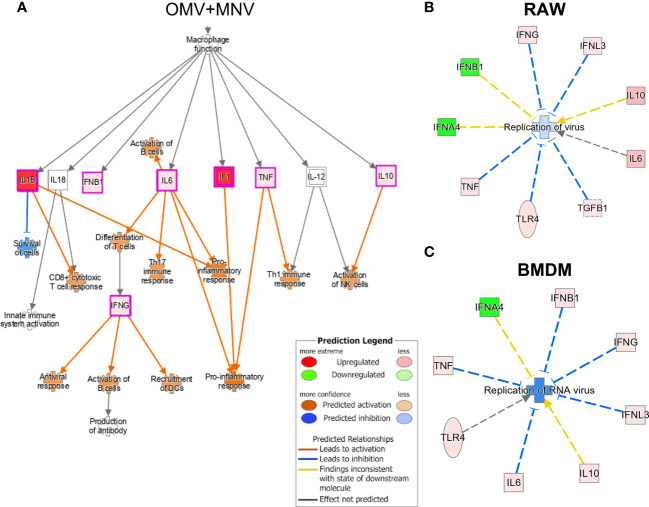
Immune pathways induced by OMVs during MNV infection as predicted by Ingenuity Pathway Analysis. Using the transcript expression data generated by Quantigene analysis of MNV and MNV + OMV (5 μg/mL) samples, potential pathways influenced by the presence of OMVs during viral infection were predicted using the IPA software. **(A)** The presence of OMVs during MNV infection was predicted to activate pro-inflammatory, Th17, and Th1 responses and also lead to the activation of both B cells and dendritic cells. **(B)** IPA analysis of transcript data also predicted OMVs induce pathways related to the inhibition of RNA virus’s replication in **(B)** RAW264.7 macrophages and **(C)** BMDMs.

Ingenuity Pathway Analysis (IPA) of the gene expression data was employed to understand the upstream and downstream impact of the observed changes in cytokine expression induced by the presence of OMVs during MNV infection. Given molecular content that comprises or is packaged within OMVs, a variety of specific bacterial metabolites and proteins may induce cytokine expression through discrete pattern recognition receptors (1, 52). Some of these compounds include lipoprotein (TLR1/TLR2, TLR2/TLR6), LPS (TLR4), flagellin (TLR5), peptidoglycan (TLR2/TLR6), and β-glucan (CLEC7A), or CpG oligonucleotide (TLR9). The presence of 5 μg/mL OMVs during MNV infection of macrophages was predicted to induce TLR4 (**Supplementary Figure 4A**). Additionally, co-inoculation of OMV+MNV in these cells was predicted to indue cytokines that would contribute to several downstream functional pathways, including CD8+ cytotoxic T cell response, differentiation of T cells, Th17 immune response, pro-inflammatory response, Th1 immune response, activation of NK cells, activation of B cells, or recruitment of dendritic cells (DCs; **Figure 8A**). These predictions indicate that OMVs may activate cellular and/or humoral immune responses aimed at the clearance of viral infection, although this has to be verified with *in vivo* studies. IPA also predicted which pathways could activate these antiviral immune responses. Specifically, LPS from the OMVs and TNF were predicted to activate the MAVS and MAPK pathways, leading to an increase in type I IFNs, activating innate immunity and contributing to antiviral immune response (**Supplementary Figure 4B**). On the other hand, during co-inoculation of macrophages with OMV+MNV, IFN-γ and Type I IFNs were predicted to activate JAK/STAT pathway, which may lead to the expression of various ISGs and subsequent antiviral immune response (**Supplementary Figure 4C**). Overall, macrophages (both RAW264.7 and BMDMs) co-inoculated with MNV and 5 μg/mL of OMVs derived from *E. cloacae*, *B. thetaiotaomicron*, or *S.* Typhimurium were predicted to cause inhibition of RNA virus replication (**Figures 8B, C**). These predicted pathways provide a path for performing targeted analysis of immune regulation of viral infection by OMVs.

The quantification of cytokines released by BMDM (**Figure 5B**) showed a similar profile to gene expression studies where MNV infection led to low IL-1β, TNFα, IFNβ, and IL-10 cytokines (53). However, adding 5 μg/mL *E. cloacae* or *B. thetaiotaomicron* OMVs during MNV infection significantly increased the production of these cytokines compared to MNV-only infection. An increase in the levels of many of these cytokines indicates an increased antiviral response, suggesting that OMVs may be stimulating these responses in macrophages. Enhancement of antiviral responses by OMVs would lead to reduced MNV replication, resulting in improved cell viability and reduced cytotoxicity, which we observed in the presence of OMVs (**Figure 4**). Therefore, OMVs may be increasing innate antiviral immune responses, which would lead to the control of MNV infection.

### MNV output after co-inoculation with bacteria and their OMVs

**Figure 9:**
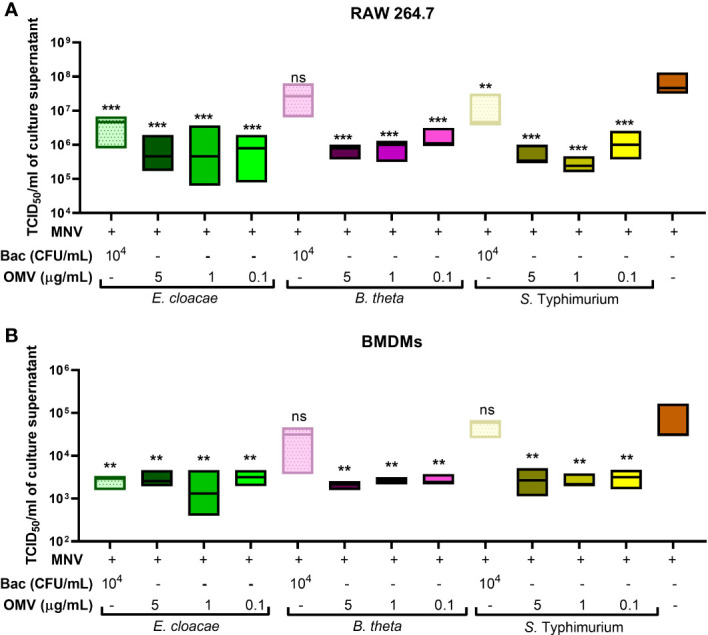
Quantification of MNV released after co-inoculation of macrophages. The supernatant was collected from **(A)** RAW264.7 cells and **(B)** BMDMs inoculated with MNV only or co-inoculated with MNV and heat inactivated bacteria (Bac) or their OMVs.TCID_50_ assay was used to measure the MNV output titer for each treatment and compared to that of MNV-only infection. Ordinary one-way ANOVA was used to analyze changes in MNV titer. (n=3; ns= not significant, **=p<0.01, ***=p<0.0001).

Since cell viability, cytotoxicity, and gene expression assays indicated that OMVs may work to suppress MNV infection, MNV replication was examined in the presence and absence of OMVs. RAW264.7 cells and BMDMs were co-inoculated with heat-inactivated bacteria + MNV, OMV + MNV or MNV and viral titers were measured at 18 hpi using TCID_50_ assay. In RAW264.7 cells, the MNV output titers in the OMV co-inoculated cells were significantly decreased compared to MNV-only infected cells (**Figure 9A**). Similarly, in BMDMs, the MNV titers cells co-inoculated with OMVs were significantly lower than that of MNV-only infected samples (**Figure 9B**). However, co-inoculation with *B. thetaiotaomicron* and *S.* Typhimurium heat-inactivated bacteria did not result in significantly decreased MNV replication, particularly in BMDMs (**Figures 9A, B**). We speculate that this may be due to an inadequate innate immune response activation by these bacteria, given the low level of transcripts observed in the multiplex mRNA quantification experiments (**Figure 5**). Together, these observations suggest that the presence of OMVs controls murine norovirus infection by promoting an antiviral response from macrophages.

## Discussion

It is significant to study the role of OMVs in MNV infection because bEVs released by commensal bacteria can communicate with distant host cells and modulate their development and functions (54–56). Under normal conditions, commensal bacteria cannot cross the intestinal barriers of mucosal and epithelial layers to enter the lamina propria, where the target cells of MNV infection reside. Furthermore, human and murine norovirus infections are characterized by only modest intestinal inflammation and a lack of epithelial barrier disruption (57). Therefore, any bacteria-mediated influence on MNV infection may be due to metabolites or bEVs released by the commensal flora (54). OMVs are known to possess multiple mechanisms to cross the epithelial barrier and interact with the underlying immune cells (58, 59). OMVs also serve as a primary means of communication between commensal bacteria and the host within the intestinal environment and thus may contribute to the modulation of immune responses during MNV infection. Interestingly, our latest study showed that exposure of intestinal commensal bacteria to noroviruses increases the production of bEVs, providing a heightened opportunity for these vesicles to impact host responses during infection (30). However, how commensal bEVs impact MNV infection and antiviral immune response is not yet known.

In a healthy gut, commensal OMVs are responsible for preserving hypo-responsiveness towards commensal bacterial antigens and maintaining intestinal homeostasis (60), but previous work has demonstrated that, during bacterial infections, OMV content may be released into the host cells (7, 61–63). Therefore, these vesicles can activate PRRs and result in a controlled immune response to eradicate the pathogen and ensure host survival.

In this study, we assessed the effect of OMVs on the host’s innate immune responses during MNV infection. We visualized and validated MNV attachment to purified OMVs in the presence and absence of the parental bacteria, indicating a robust interaction between the virus and the vesicles. Considering this, we speculated that MNV may migrate with OMVs through the epithelial barrier, leading to both virus and vesicle being present during MNV infection. MNV migrates from the intestinal lumen to the lamina propria using M cells (27), and OMVs may be taken up similarly during infection allowing the virus and bEVs to access the gut-associated lymphoid tissue where MNV replicates robustly (54). Additionally, since MNV infection in the mouse leads to an increased production of bEVs (30), even in the absence of MNV-OMV complexes, the increased presence of vesicles during infection may allow for increased concentrations of bEVs at target sites during MNV replication. However, whether co-exposure of virus and bEVs to target cells *in vivo* occurs as an MNV-vesicle complex or separately is currently being investigated.

Given the established ability of OMVs to modulate host immune responses during infection, we investigated the impact OMVs from *E. cloacae*, *B. thetaiotaomicron*, and *S.* Typhimurium on antiviral immune responses during MNV infection of macrophages. *S.* Typhimurium was used to explore the effects of OMVs derived from an intestinal pathogen compared to those produced by commensal bacteria. The presence of OMVs during MNV infection lowered the MNV-induced cytotoxicity of the cells, indicating that cellular health was improved, and viral replication may be suppressed. Innate immune responses against noroviruses play an important role in decreasing viral replication which reduce virus-induced cytopathological effect. Given this and the ability of OMVs to regulate these responses (2, 3), we set out to determine if the lowering of cytotoxicity in the presence of OMVs was due to changes in immune response. We found that, other than type I IFNs, all other cytokines and TLR-4 is expressed in low quantity during MNV infection. It was interesting to note that most of these significant changes occurred only in the presence of higher OMV concentrations (5 μg/mL) suggesting an association between the increased production of OMVs during murine norovirus infection (30) and the induction of innate immune responses. Although commensal OMVs regularly maintain intestinal homeostasis, they are also known to have altered functions during infection and diseases to ensure host survival (60, 61, 63–65).

Since cellular cytotoxicity assays and cytokine expression indicated that viral replication was suppressed, MNV titers in the presence and absence of OMVs was also measured. These results showed viral titers were significantly reduced in OMV treated cells, supporting the idea that these vesicles are involved in the control of MNV replication. Collectively, this work demonstrates that the increasing presence of OMVs can induce antiviral immune responses during MNV infection which correlates with reduced cellular cytotoxicity and control of viral replication. Both type I and type II IFN responses are essential for early inhibition of the MNV infection cycle by inhibiting translation (48). However, MNV exercises multiple ways to suppress these responses by preventing translation of host cytokines. The control of host translational machinery also helps MNV to replicate efficiently, and MNV prevents cellular mRNA translation through: 1) MNV phosphorylation of eIF4E *via* MAPK pathway where eIF4E is sent to polysomes which alters the translational state of cellular mRNAs (53), 2) NS6 and cellular caspases inhibit ISG translation during MNV infection, and 3) the viral protease cleaves PAPB which facilitates the binding of eIF4G to poly A tail of cellular mRNAs (66) and MNV phosphorylates eIF2a *via* protein kinase R, thus suppressing host protein translation (67). Therefore, for OMVs to inhibit MNV replication, they must counteract MNV suppression of inflammatory responses or induce antiviral responses through pathways that are not controlled by MNV. Interestingly, it has been recently shown that OMVs from commensal bacteria can induce STING-mediated inflammatory pathways leading to control of systemic infection of both RNA and DNA viruses (68). Moreover, while artificial induction of STING responses can lead to control of MNV replication, MNV infection itself does not influence STING expression (69). Based on these publications and our findings reported herein, we speculate and are currently testing the hypothesis that bEVs provide the host with a mechanism for controlling enteric viral infection. Through increased bEV production during MNV infection, these vesicles induce inflammatory pathways that cannot be suppressed by viral proteins, ultimately allowing for control of MNV replication. This study is the first to identify immune modulation by commensal OMVs taking place during enteric viral infection, and to demonstrate a link between these vesicles and infection control.

## Data availability statement

The original contributions presented in the study are included in the article/**Supplementary Material**. Further inquiries can be directed to the corresponding author.

## Ethics statement

The animal study was reviewed and approved by University of Florida Institutional Animal Care and Use Committee.

## Author contributions

MJ, ME, and SB contributed to the conception and design of the study. MJ, ME, SB, and GZ contributed to method design and implementation. SB, GZ, JB, HS, and ADM performed experiments included in this study. MJ, SB, and GZ contributed to data analysis and validation. MJ, ME, and SB contributed to supervision of the study. MJ and ME acquired funding for the project and MJ oversaw project administration. SB wrote the first draft of the manuscript. MJ, ME, SB, and GZ contributed sections or performed extensive editing of the manuscript. All authors contributed to manuscript revision, read and approved the submitted version.

## Funding

This work was supported in part by grants R21AI140012 and R03AI135610 from the National Institutes of Health, the Early Career Scientist Award provided by the Institute of Food and Agricultural Sciences at the University of Florida, and the USDA National Institute of Food and Agriculture, Hatch Project 1015632.

## Acknowledgments

We acknowledge the Electron-Microscopy and Bio-imaging Core, Interdisciplinary Center for Biotechnology Research (ICBR), University of Florida, for their assistance in providing electron microscopic images. We thank Dr. Fernanda Rocha and Dr. Frank Gibson for assistance with the MagPix instrument, and Dr. Sarah Stuart Chewning and Jim Giron from the Protein and Cellular Analysis team of Thermo Fisher Scientific for QuantiGene training.

## Conflict of interest

The authors declare that the research was conducted in the absence of any commercial or financial relationships that could be construed as a potential conflict of interest.

## Publisher’s note

All claims expressed in this article are solely those of the authors and do not necessarily represent those of their affiliated organizations, or those of the publisher, the editors and the reviewers. Any product that may be evaluated in this article, or claim that may be made by its manufacturer, is not guaranteed or endorsed by the publisher.

## References

[1] Nagakubo T Nomura N Toyofuku M. Cracking open bacterial membrane vesicles. Front Microbiol (2019) 10:3026. doi: 10.3389/fmicb.2019.03026 32038523 PMC6988826

[2] Ellis TN Leiman SA Kuehn MJ. Naturally produced outer membrane vesicles from pseudomonas aeruginosa elicit a potent innate immune response *via* combined sensing of both lipopolysaccharide and protein components. Infect Immun (2010) 78(9):3822–31. doi: 10.1128/IAI.00433-10 PMC2937433 20605984

[3] Kim CU Eo S Lee P Kim SH Kim YS Kim DJ. Pretreatment of outer membrane vesicle and subsequent infection with influenza virus induces a long-lasting adaptive immune response against broad subtypes of influenza virus. Microbes Infect (2022) 24(1):104878. doi: 10.1016/j.micinf.2021.104878 34384869

[4] Baker SM Settles EW Davitt C Gellings P Kikendall N Hoffmann J. Burkholderia pseudomallei OMVs derived from infection mimicking conditions elicit similar protection to a live-attenuated vaccine. NPJ Vaccines (2021) 6(1):18. doi: 10.1038/s41541-021-00281-z 33514749 PMC7846723

[5] Prior JT Davitt C Kurtz J Gellings P McLachlan JB Morici LA. Bacterial-derived outer membrane vesicles are potent adjuvants that drive humoral and cellular immune responses. Pharmaceutics (2021) 13(2). doi: 10.3390/pharmaceutics13020131 PMC7909432 33498352

[6] Chatterjee D Chaudhuri K. Vibrio cholerae O395 outer membrane vesicles modulate intestinal epithelial cells in a NOD1 protein-dependent manner and induce dendritic cell-mediated Th2/Th17 cell responses. J Biol Chem (2013) 288(6):4299–309. doi: 10.1074/jbc.M112.408302 PMC3567681 23275338

[7] Fábrega MJ Aguilera L Giménez R Varela E Alexandra Cañas M Antolín M. Activation of immune and defense responses in the intestinal mucosa by outer membrane vesicles of commensal and probiotic escherichia coli strains. Front Microbiol (2016) 7:705. doi: 10.3389/fmicb.2016.00705 27242727 PMC4863414

[8] Bae EH Seo SH Kim CU Jang MS Song MS Lee TY. Bacterial outer membrane vesicles provide broad-spectrum protection against influenza virus infection *via* recruitment and activation of macrophages. J Innate Immun (2019) 11(4):316–29. doi: 10.1159/000494098 PMC6738265 30844806

[9] Wang S Gao J Wang Z. Outer membrane vesicles for vaccination and targeted drug delivery. Wiley Interdiscip Rev Nanomed Nanobiotechnol (2019) 11(2):e1523. doi: 10.1002/wnan.1523 29701017 PMC6203682

[10] Carvalho AL Fonseca S Miquel-Clopés A Cross K Kok KS Wegmann U. Bioengineering commensal bacteria-derived outer membrane vesicles for delivery of biologics to the gastrointestinal and respiratory tract. J Extracell Vesicles (2019) 8(1):1632100. doi: 10.1080/20013078.2019.1632100 31275534 PMC6598475

[11] Zurita ME Wilk MM Carriquiriborde F Bartel E Moreno G Misiak A. A pertussis outer membrane vesicle-based vaccine induces lung-resident memory CD4 T cells and protection against. Front Cell Infect Microbiol (2019) 9:125. doi: 10.3389/fcimb.2019.00125 31106160 PMC6498398

[12] Guebre-Xabier M Patel N Tian JH Zhou B Maciejewski S Lam K. NVX-CoV2373 vaccine protects cynomolgus macaque upper and lower airways against SARS-CoV-2 challenge. Vaccine (2020) 38(50):7892–6. doi: 10.1016/j.vaccine.2020.10.064 PMC7584426 33139139

[13] Lee BJ Kwon HI Kim EH Park SJ Lee SH Choi YK. Assessment of mOMV adjuvant efficacy in the pathogenic H1N1 influenza virus vaccine. Clin Exp Vaccine Res (2014) 3(2):194–201. doi: 10.7774/cevr.2014.3.2.194 25003093 PMC4083072

[14] Unal CM Schaar V Riesbeck K. Bacterial outer membrane vesicles in disease and preventive medicine. Semin Immunopathol (2011) 33(5):395–408. doi: 10.1007/s00281-010-0231-y 21153593

[15] Koo HL Neill FH Estes MK Munoz FM Cameron A DuPont HL. Noroviruses: The most common pediatric viral enteric pathogen at a Large university hospital after introduction of rotavirus vaccination. J Pediatr Infect Dis Soc (2013) 2(1):57–60. doi: 10.1093/jpids/pis070 PMC3656546 23687584

[16] Atmar RL Opekun AR Gilger MA Estes MK Crawford SE Neill FH. Norwalk Virus shedding after experimental human infection. Emerg Infect Dis (2008) 14(10):1553–7. doi: 10.3201/eid1410.080117 PMC2609865 18826818

[17] Ettayebi K Crawford SE Murakami K Broughman JR Karandikar U Tenge VR. Replication of human noroviruses in stem cell-derived human enteroids. Science (2016) 353(6306):1387–93. doi: 10.1126/science.aaf5211 PMC5305121 27562956

[18] Davis A Cortez V Grodzki M Dallas R Ferrolino J Freiden P. Infectious norovirus is chronically shed by immunocompromised pediatric hosts. Viruses (2020) 12(6). doi: 10.3390/v12060619 PMC7354526 32516960

[19] Jones MK Watanabe M Zhu S Graves CL Keyes LR Grau KR. Enteric bacteria promote human and mouse norovirus infection of b cells. Science (2014) 346(6210):755–9. doi: 10.1126/science.1257147 PMC4401463 25378626

[20] Todd KV Tripp RA. Vero cells as a mammalian cell substrate for human norovirus. Viruses (2020) 12(4). doi: 10.3390/v12040439 PMC7232407 32295124

[21] Karandikar UC Crawford SE Ajami NJ Murakami K Kou B Ettayebi K. Detection of human norovirus in intestinal biopsies from immunocompromised transplant patients. J Gen Virol (2016) 97(9):2291–300. doi: 10.1099/jgv.0.000545 PMC5756488 27412790

[22] Karst SM Wobus CE Lay M Davidson J Virgin HW4. STAT1-dependent innate immunity to a Norwalk-like virus. Science (2003) 299(5612):1575–8. doi: 10.1126/science.1077905 12624267

[23] Mumphrey SM Changotra H Moore TN Heimann-Nichols ER Wobus CE Reilly MJ. Murine norovirus 1 infection is associated with histopathological changes in immunocompetent hosts, but clinical disease is prevented by STAT1-dependent interferon responses. J Virol (2007) 81(7):3251–63. doi: 10.1128/JVI.02096-06 PMC1866040 17229692

[24] Zhu S Regev D Watanabe M Hickman D Moussatche N Jesus DM. Identification of immune and viral correlates of norovirus protective immunity through comparative study of intra-cluster norovirus strains. PloS Pathog (2013) 9(9):e1003592. doi: 10.1371/journal.ppat.1003592 24039576 PMC3764223

[25] Zhu S Jones MK Hickman D Han S Reeves W Karst SM. Norovirus antagonism of b-cell antigen presentation results in impaired control of acute infection. Mucosal Immunol (2016) 9(6):1559–70. doi: 10.1038/mi.2016.15 PMC5035161 27007673

[26] Bragazzi Cunha J Wobus CE. Select membrane proteins modulate MNV-1 infection of macrophages and dendritic cells in a cell type-specific manner. Virus Res (2016) 222:64–70. doi: 10.1016/j.virusres.2016.06.001 27264433 PMC6053272

[27] Gonzalez-Hernandez MB Liu T Blanco LP Auble H Payne HC Wobus CE. Murine norovirus transcytosis across an *in vitro* polarized murine intestinal epithelial monolayer is mediated by m-like cells. J Virol (2013) 87(23):12685–93. doi: 10.1128/JVI.02378-13 PMC3838167 24049163

[28] Perry JW Taube S Wobus CE. Murine norovirus-1 entry into permissive macrophages and dendritic cells is pH-independent. Virus Res (2009) 143(1):125–9. doi: 10.1016/j.virusres.2009.03.002 PMC2687405 19463729

[29] Madrigal JL Bhar S Hackett S Engelken H Joseph R Keyhani NO. Attach me if you can: Murine norovirus binds to commensal bacteria and fungi. Viruses (2020) 12(7). doi: 10.3390/v12070759 PMC7412252 32674489

[30] Mosby CA Bhar S Phillips MB Edelmann MJ Jones MK. Interaction with mammalian enteric viruses alters outer membrane vesicle production and content by commensal bacteria. J Extracell Vesicles (2022) 11(1):e12172. doi: 10.1002/jev2.12172 34981901 PMC8725172

[31] Mozaheb N Mingeot-Leclercq MP. Membrane vesicle production as a bacterial defense against stress. Front Microbiol (2020) 11:600221. doi: 10.3389/fmicb.2020.600221 33362747 PMC7755613

[32] Bos J Cisneros LH Mazel D. Real-time tracking of bacterial membrane vesicles reveals enhanced membrane traffic upon antibiotic exposure. Sci Adv (2021) 7(4):1830–1836. doi: 10.1126/sciadv.abd1033 PMC7817102 33523924

[33] Gamalier JP Silva TP Zarantonello V Dias FF Melo RC. Increased production of outer membrane vesicles by cultured freshwater bacteria in response to ultraviolet radiation. Microbiol Res (2017) 194:38–46. doi: 10.1016/j.micres.2016.08.002 27938861

[34] Grau KR Zhu S Peterson ST Helm EW Philip D Phillips M. The intestinal regionalization of acute norovirus infection is regulated by the microbiota *via* bile acid-mediated priming of type III interferon. Nat Microbiol (2020) 5(1):84–92. doi: 10.1038/s41564-019-0602-7 31768030 PMC6925324

[35] Li N Ma WT Pang M Fan QL Hua JL. The commensal microbiota and viral infection: A comprehensive review. Front Immunol (2019) 10:1551. doi: 10.3389/fimmu.2019.01551 31333675 PMC6620863

[36] Karst SM. The influence of commensal bacteria on infection with enteric viruses. Nat Rev Microbiol (2016) 14(4):197–204. doi: 10.1038/nrmicro.2015.25 26853118 PMC5198578

[37] Roth AN Grau KR Karst SM. Diverse mechanisms underlie enhancement of enteric viruses by the mammalian intestinal microbiota. Viruses (2019) 11(8). doi: 10.3390/v11080760 PMC6722614 31426458

[38] Woods Acevedo MA Pfeiffer JK. Microbiota-immune system interactions and enteric virus infection. Curr Opin Virol (2021) 46:15–9. doi: 10.1016/j.coviro.2020.08.005 PMC7933313 32898729

[39] Almand EA Moore MD Outlaw J Jaykus LA. Human norovirus binding to select bacteria representative of the human gut microbiota. PloS One (2017) 12(3):e0173124. doi: 10.1371/journal.pone.0173124 28257478 PMC5336261

[40] Zhang S Kingsley RA Santos RL Andrews-Polymenis H Raffatellu M Figueiredo J. Molecular pathogenesis of salmonella enterica serotype typhimurium-induced diarrhea. Infect Immun (2003) 71(1):1–12. doi: 10.1128/IAI.71.1.1-12.2003 12496143 PMC143292

[41] Bhar S Edelmann MJ Jones MK. Characterization and proteomic analysis of outer membrane vesicles from a commensal microbe, enterobacter cloacae. J Proteomics (2020), 231:103994. doi: 10.1016/j.jprot.2020.103994 33007464

[42] Turner L Bitto NJ Steer DL Lo C D'Costa K Ramm G. Outer membrane vesicle size determines their mechanisms of host cell entry and protein content. Front Immunol (2018) 9:1466. doi: 10.3389/fimmu.2018.01466 30013553 PMC6036113

[43] Shen Y Giardino Torchia ML Lawson GW Karp CL Ashwell JD Mazmanian SK. Outer membrane vesicles of a human commensal mediate immune regulation and disease protection. Cell Host Microbe (2012) 12(4):509–20. doi: 10.1016/j.chom.2012.08.004 PMC3895402 22999859

[44] Cañas MA Fábrega MJ Giménez R Badia J Baldomà L. Outer membrane vesicles from probiotic and commensal. Front Microbiol (2018) 9:498. doi: 10.3389/fmicb.2018.00498 29616010 PMC5869251

[45] Alpdundar Bulut E Bayyurt Kocabas B Yazar V Aykut G Guler U Salih B. Human gut commensal membrane vesicles modulate inflammation by generating M2-like macrophages and myeloid-derived suppressor cells. J Immunol (2020) 205(10):2707–18. doi: 10.4049/jimmunol.2000731 33028617

[46] Baldridge MT Nice TJ McCune BT Yokoyama CC Kambal A Wheadon M. Commensal microbes and interferon-λ determine persistence of enteric murine norovirus infection. Science (2015) 347(6219):266–9. doi: 10.1126/science.1258025 PMC4409937 25431490

[47] Baldridge MT Turula H Wobus CE. Norovirus regulation by host and microbe. Trends Mol Med (2016) 22(12):1047–59. doi: 10.1016/j.molmed.2016.10.003 PMC5135607 27887808

[48] Changotra H Jia Y Moore TN Liu G Kahan SM Sosnovtsev SV. Type I and type II interferons inhibit the translation of murine norovirus proteins. J Virol (2009) 83(11):5683–92. doi: 10.1128/JVI.00231-09 PMC2681988 19297466

[49] Nice TJ Robinson BA Van Winkle JA. The role of interferon in persistent viral infection: Insights from murine norovirus. Trends Microbiol (2018) 26(6):510–24. doi: 10.1016/j.tim.2017.10.010 PMC5957778 29157967

[50] Ahmadi Badi S Khatami SH Irani SH Siadat SD. Induction effects of bacteroides fragilis derived outer membrane vesicles on toll like receptor 2, toll like receptor 4 genes expression and cytokines concentration in human intestinal epithelial cells. Cell J (2019) 21(1):57–61. doi: 10.22074/cellj.2019.5750 30507089 PMC6275420

[51] Sundin J Rangel I Repsilber D Brummer RJ. Cytokine response after stimulation with key commensal bacteria differ in post-infectious irritable bowel syndrome (PI-IBS) patients compared to healthy controls. PloS One (2015) 10(9):e0134836. doi: 10.1371/journal.pone.0134836 26366730 PMC4569289

[52] Mancini F Rossi O Necchi F Micoli F. OMV vaccines and the role of TLR agonists in immune response. Int J Mol Sci (2020) 21(12). doi: 10.3390/ijms21124416 PMC7352230 32575921

[53] Royall E Doyle N Abdul-Wahab A Emmott E Morley SJ Goodfellow I. Murine norovirus 1 (MNV1) replication induces translational control of the host by regulating eIF4E activity during infection. J Biol Chem (2015) 290(8):4748–58. doi: 10.1074/jbc.M114.602649 PMC4335213 25561727

[54] Grau KR Roth AN Zhu S Hernandez A Colliou N DiVita BB. The major targets of acute norovirus infection are immune cells in the gut-associated lymphoid tissue. Nat Microbiol (2017) 2(12):1586–91. doi: 10.1038/s41564-017-0057-7 PMC5705318 29109476

[55] Peng Y Yin S Wang M. Extracellular vesicles of bacteria as potential targets for immune interventions. Hum Vaccin Immunother (2021) 17(3):897–903. doi: 10.1080/21645515.2020.1799667 32873124 PMC7993133

[56] Macia L Nanan R Hosseini-Beheshti E Grau GE. Host- and microbiota-derived extracellular vesicles, immune function, and disease development. Int J Mol Sci (2019) 21(1). doi: 10.3390/ijms21010107 PMC6982009 31877909

[57] Blacklow NR Dolin R Fedson DS Dupont H Northrup RS Hornick RB. Acute infectious nonbacterial gastroenteritis: etiology and pathogenesis. Ann Intern Med (1972) 76(6):993–1008. doi: 10.7326/0003-4819-76-6-993 4554885

[58] Stentz R Carvalho AL Jones EJ Carding SR. Fantastic voyage: the journey of intestinal microbiota-derived microvesicles through the body. Biochem Soc Trans (2018) 46(5):1021–7. doi: 10.1042/BST20180114 PMC6195637 30154095

[59] Kaparakis-Liaskos M Ferrero RL. Immune modulation by bacterial outer membrane vesicles. Nat Rev Immunol (2015) 15(6):375–87. doi: 10.1038/nri3837 25976515

[60] Durant L Stentz R Noble A Brooks J Gicheva N Reddi D. Bacteroides thetaiotaomicron-derived outer membrane vesicles promote regulatory dendritic cell responses in health but not in inflammatory bowel disease. Microbiome (2020) 8(1):88. doi: 10.1186/s40168-020-00868-z 32513301 PMC7282036

[61] Cañas MA Giménez R Fábrega MJ Toloza L Baldomà L Badia J. Outer membrane vesicles from the probiotic escherichia coli nissle 1917 and the commensal ECOR12 enter intestinal epithelial cells *via* clathrin-dependent endocytosis and elicit differential effects on DNA damage. PloS One (2016) 11(8):e0160374. doi: 10.1371/journal.pone.0160374 27487076 PMC4972321

[62] Nicholson JK Holmes E Kinross J Burcelin R Gibson G Jia W. Host-gut microbiota metabolic interactions. Science (2012) 336(6086):1262–7. doi: 10.1126/science.1223813 22674330

[63] Engevik MA Danhof HA Ruan W Engevik AC Chang-Graham AL Engevik KA. Secretes outer membrane vesicles and promotes intestinal inflammation. mBio (2021) 12(2):e2706-20. doi: 10.1128/mBio.02706-20 PMC8092269 33653893

[64] Gul L Modos D Fonseca S Madgwick M Thomas JP Sudhakar P. Extracellular vesicles produced by the human commensal gut bacterium bacteroides thetaiotaomicron affect host immune pathways in a cell-type specific manner that are altered in inflammatory bowel disease. J Extracell Vesicles (2022) 11(1):e12189. doi: 10.1002/jev2.12189 35064769 PMC8783345

[65] Tiku V Tan MW. Host immunity and cellular responses to bacterial outer membrane vesicles. Trends Immunol (2021) 42(11):1024–36. doi: 10.1016/j.it.2021.09.006 34635395

[66] Emmott E Sorgeloos F Caddy SL Vashist S Sosnovtsev S Lloyd R. Norovirus-mediated modification of the translational landscape *via* virus and host-induced cleavage of translation initiation factors. Mol Cell Proteomics (2017) 16(4 suppl 1):S215–29. doi: 10.1074/mcp.M116.062448 PMC5393397 28087593

[67] Fritzlar S Aktepe TE Chao YW Kenney ND McAllaster MR Wilen CB. Mouse norovirus infection arrests host cell translation uncoupled from the stress granule-PKR-eIF2α axis. mBio (2019) 10(3) e00960-19. doi: 10.1128/mBio.00960-19 31213553 PMC6581855

[68] Erttmann SF Swacha P Aung KM Brindefalk B Jiang H Härtlova A. The gut microbiota prime systemic antiviral immunity *via* the cGAS-STING-IFN-I axis. Immunity (2022) 55(5):847–861.e10. doi: 10.1016/j.immuni.2022.04.006 35545033

[69] Yu P Miao Z Li Y Bansal R Peppelenbosch MP Pan Q. cGAS-STING effectively restricts murine norovirus infection but antagonizes the antiviral action of n-terminus of RIG-I in mouse macrophages. Gut Microbes (2021) 13(1):1959839. doi: 10.1080/19490976.2021.1959839 34347572 PMC8344765

