# Supplementary material for: Bacterial extracellular vesicles control murine norovirus infection through modulation of antiviral immune responses
Source: Front Immunol. 2022 Aug 4;13:909949. doi: 10.3389/fimmu.2022.909949 (PMC9386532; doi:10.3389/fimmu.2022.909949)
Supplement: Supplementary file 1 [file DataSheet_1.docx]

Supplementary Material

# Supplementary Figures and Tables

## Supplementary Figures


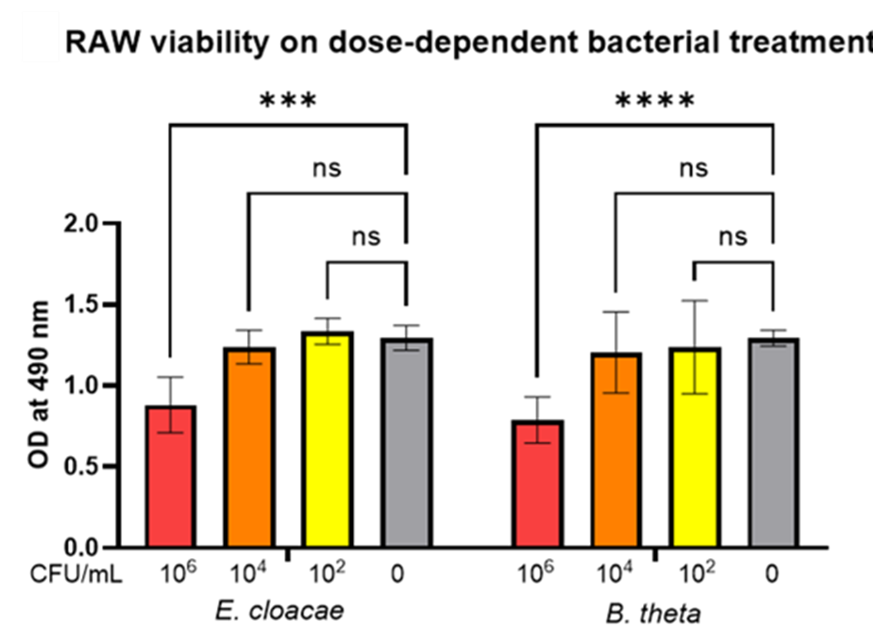


**Supplementary Figure 1.** Macrophage viability after incubation with commensal bacteria. RAW 264.7 cells were incubated with increasing concentrations of heat killed (65°C) *E. cloacae* or *B. thetaiotaomicron*. Cell viability of was measured 18 hours post-treatment with using MTS assay. One-way ANOVA was used to determine statistical significance. (n=3; ns=not significant, ***=p<0.001, ****=p<0.0001).


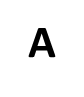


**Supplementary Figure 2.** Impact of *S.* Typhimurium on viability and cytotoxicity of RAW 264.7 macrophages at 18 hrs post co-infection. A) Cell viability on treatment with *S.* Typhimurium was measured 18 hours post treatment with OMVs only, MNV only, or MNV+OMV using MTS assay (n=3 for all experiments). B) LDH released 18 hours post infection (hpi) on treatment with PBS, OMVs only, MNV only, and OMV-MNV co-infection, was measured using spectrophotometry (n=3) (*=p<0.05, **=p<0.01, ***=p<0.001, ****=p<0.001).


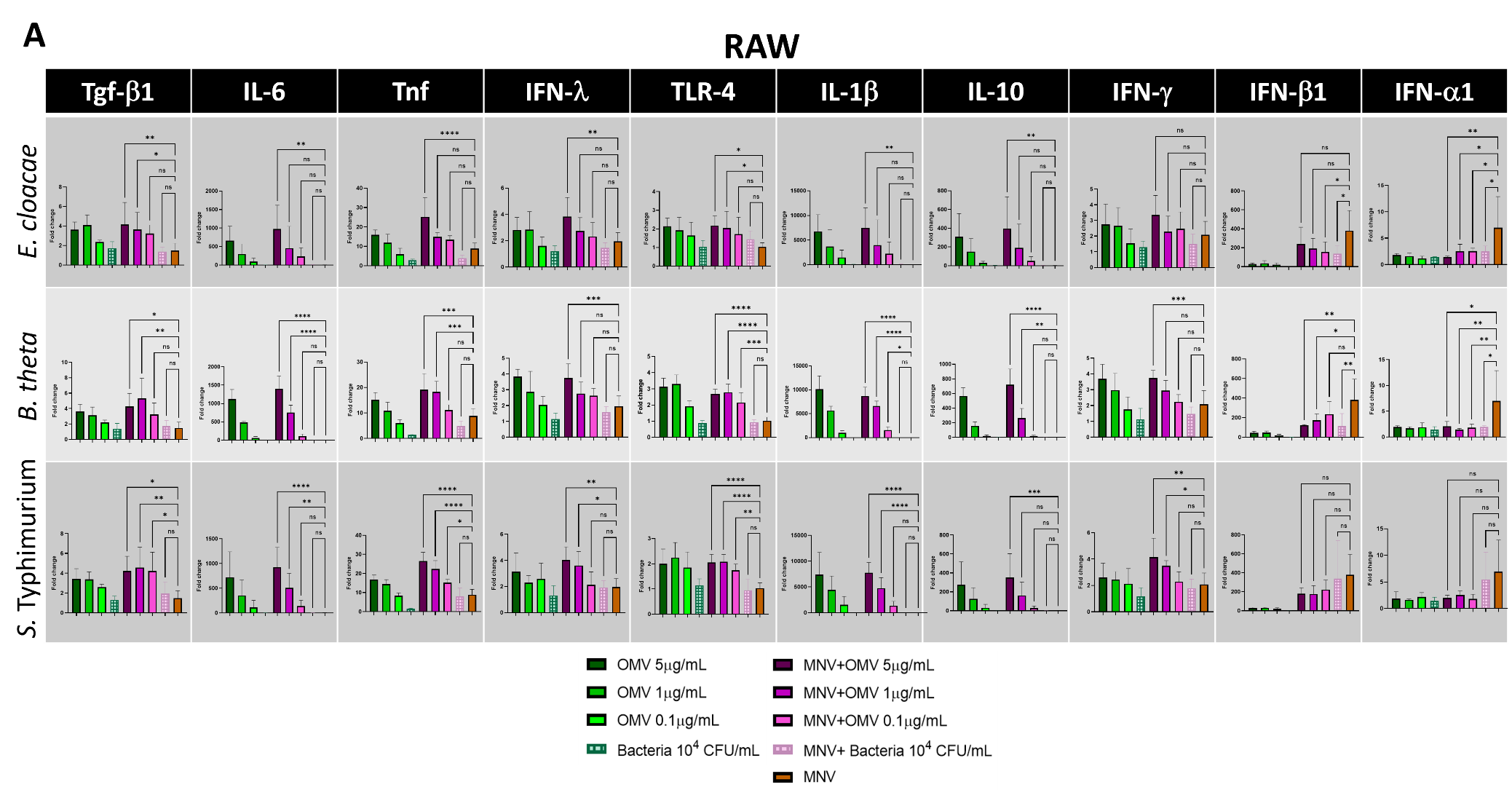


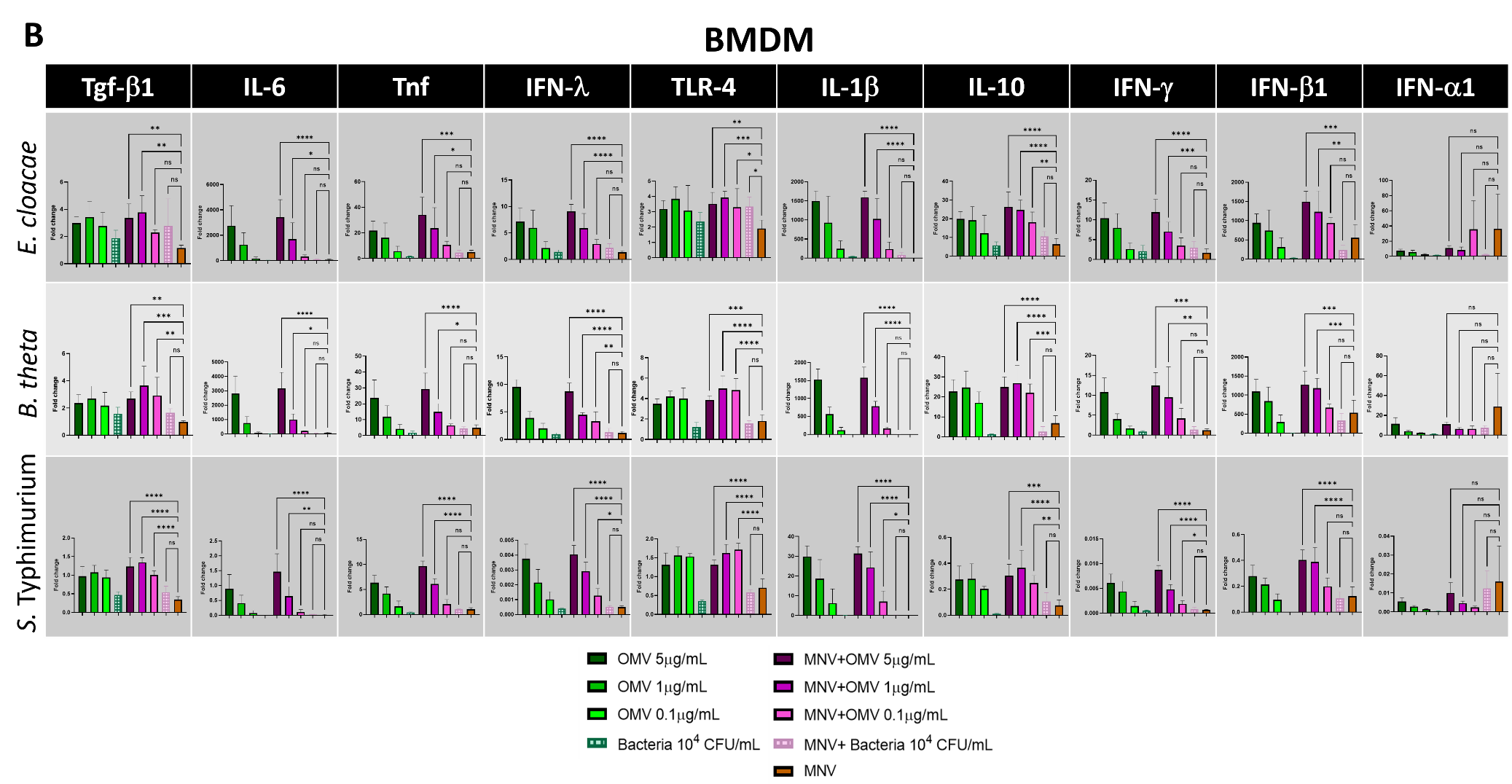
**Supplementary Figure 3.** Changes in cytokine transcript expression after co-infections. The fold change of treated samples compared to mock analyzed from QuantiGene mRNA quantification assay was graphed for A) RAW264.7 cells and B) BMDMs for TGF-β, IL-6, TNFα, IFN-λ, TLR4, IL-1β, IL-10, IGN-γ, IFN-β1, IFN-α1 transcripts (ns= not significant, *=p<0.05, **=p<0.01, ***=p<0.001, ****=p<0.0001).


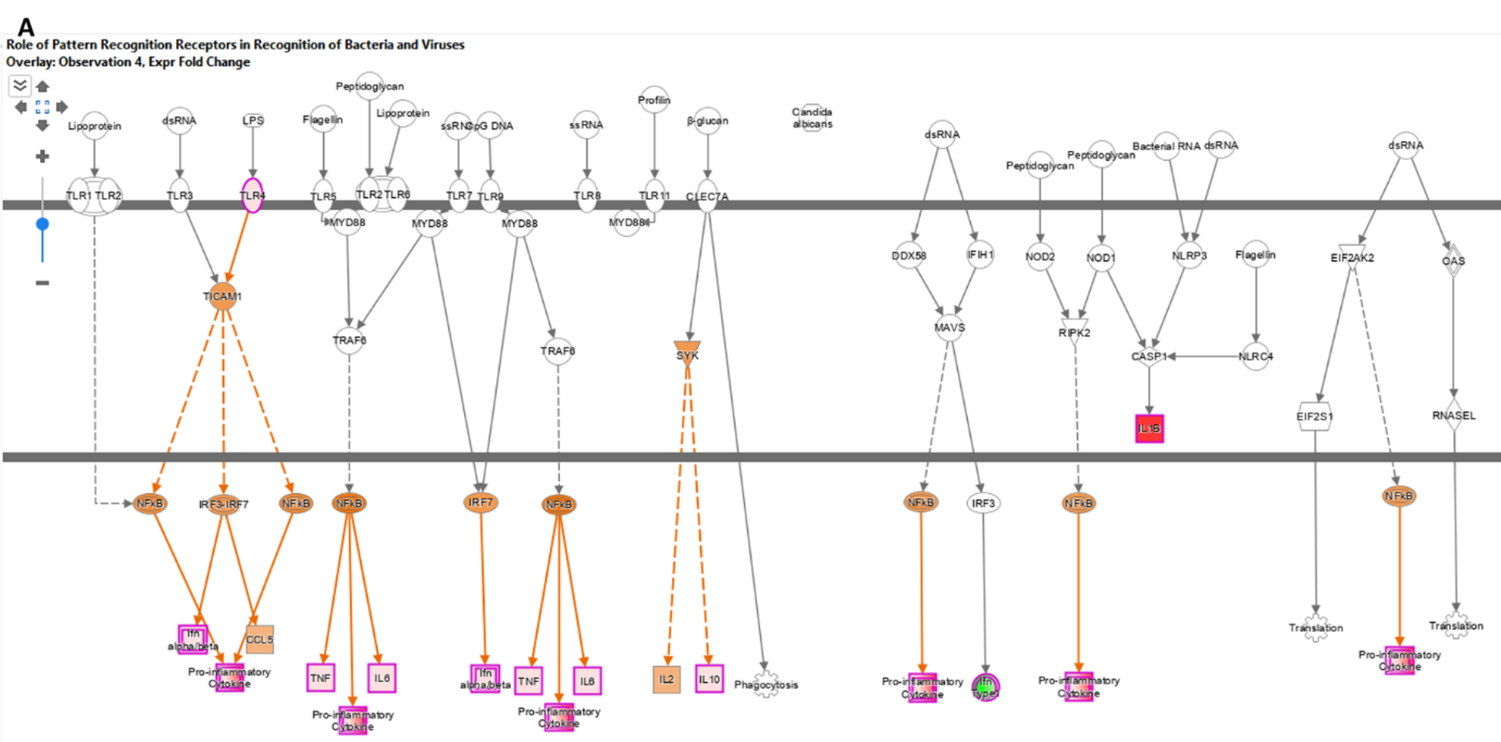


**A**


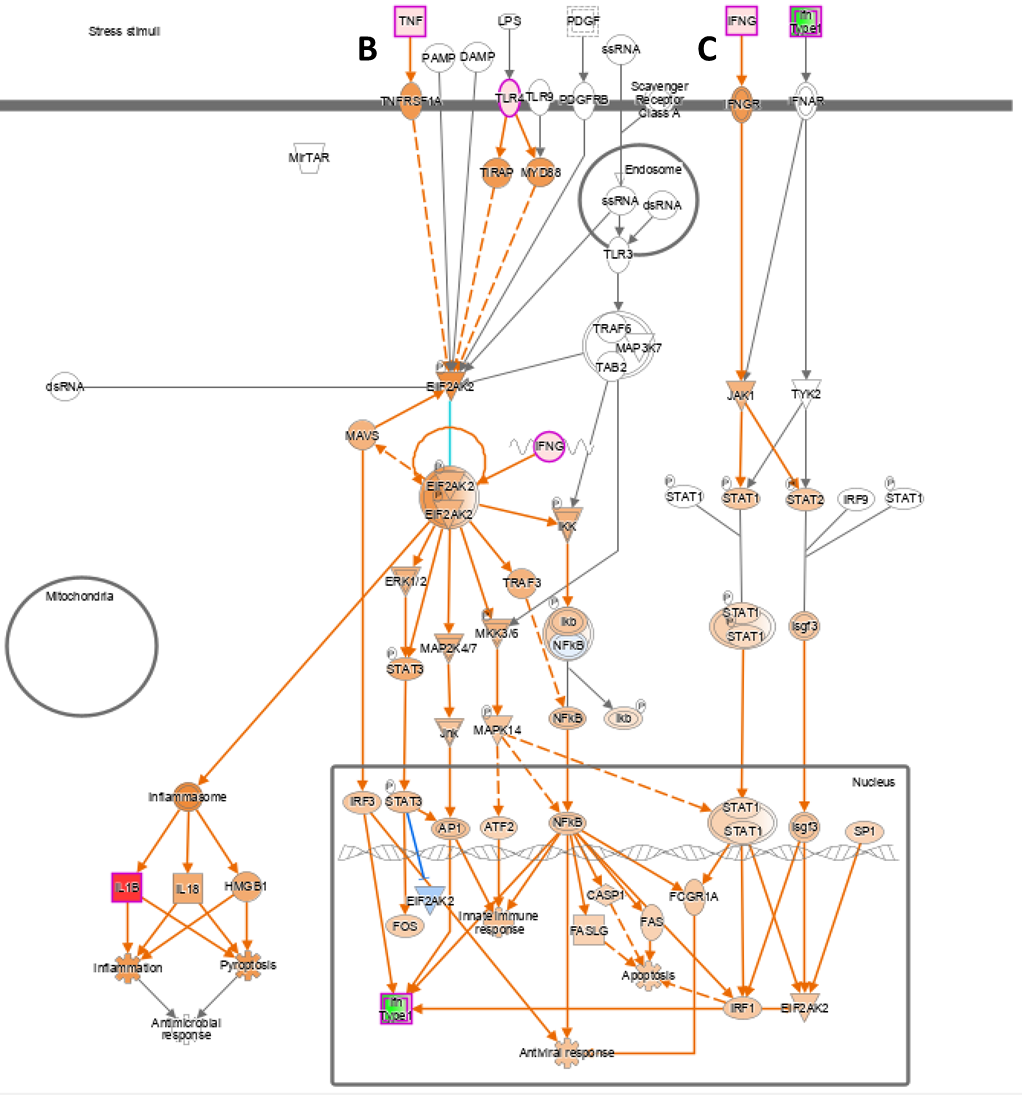


**B**

**C**


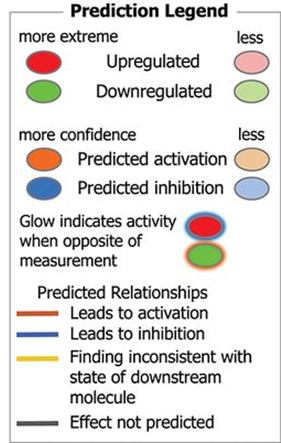


**Supplementary Figure 4.** Predicted upstream and downstream pathways based on cytokine expression analysis. Transcripts for cytokines expressed by macrophages when co-inoculated with OMV+MNV were measured using Quantigene. Ingenuity Pathway Analysis was used for pathway prediction. A) Upstream regulators most predicted to activate TLRs leading to release of the cytokines quantified in QuantiGene. Predicted activation of B) MAPK pathways and C) JAK/STAT pathway potentially leading to inflammation, pyroptosis, and anti-viral response.

## Supplementary Tables

**Supplementary Table 1.** RT-qPCR primer sequences for MNV and ISG detection

| Gene | Forward primer | Reverse primer |
| --- | --- | --- |
| MNV-1 | GTGCGCAACACAGAGAAACG | CGGGCTGAGCTTCCTGC |
| IRF1 | CAGAGGAAAGAGAGAAAGTCC | CACACGGTGACAGTGCTGG |
| ISG15 | TGACGCAGACTGTAGACACG | TGGGGCTTTAGGCCATACTC |
| MX1 | GGGGAGGAAATAGAGAAAATGAT | GTTTACAAAGGGCTTGCTTGCT |
| IFIT1 | CCATAGCGGAGGTGAATATC | GGCAGGACAATGTGCAAGAA |
| GAPDH | CGACTTCAACAGCAACTCCCACTCTTCC | TGGGTGGTCCAGGGTTTCTTACTCCTT |

**Supplementary Table 2.** Target-bead association in QuantiGene Plex reagent system.

| Bead number | Target symbol | Target name |
| --- | --- | --- |
| 12 | TGFβ1 | Transforming growth factor, beta 1 |
| 13 | IL6 | Interleukin 6 |
| 19 | PpiB | Peptidylprolyl isomerase B |
| 25 | HPRT1 | Hypoxanthine phosphoribosyltransferase 1 |
| 26 | TNF | Tumor necrosis factor |
| 28 | IL28β | Interleukin 28 |
| 29 | TLR4 | Toll-like receptor 4 |
| 34 | IL1β | Interleukin 1 beta |
| 43 | IL10 | Interleukin 10 |
| 46 | IFNβ1 | Interferon beta 1 |
| 55 | IFNα1 | Interferon alpha family, gene 1 |
| 56 | IFNγ | Interferon gamma |

**Supplementary Table 3.** Bead probe set information.

| Accession number | Target symbol | Other names | Sequence length | Probe set region | Specificity |
| --- | --- | --- | --- | --- | --- |
| NM_011577 | TGFβ1 | TGFβ; TGFβ-1 | 2094 | 918-1536 | Mouse TGFβ1 |
| NM_031168 | IL6 | IL-6 | 1087 | 249-1066 | Mouse IL6 |
| NM_011149 | PpiB | Cphn2; Cphn-2; CyP-20b | 979 | 207-588 | Mouse PpiB |
| NM_000194 | HPRT1 | HPRT; HGPRT | 1435 | 102-646 | Human HPRT1 |
| NM_013693 | TNF | DIF; TNFalpha; TNFα; TNFSF2; Tnfsf1α; TNF-alpha | 1619 | 590-1163 | Mouse TNF |
| NM_177396 | IL28β | IFN-λ, IFN-lambda | 582 | 2-503 | Mouse IFNλ |
| NM_021297 | TLR4 | Lps; Ly87; Ran/M1; Rasl2-8 | 3847 | 390-1148 | Mouse TLR4 |
| NM_008361 | IL1β | IL-1β; IL-1beta | 1328 | 414-950 | Mouse IL1β |
| NM_010548 | IL10 | CSIF; IL-10 | 1306 | 322-759 | Mouse IL10 |
| NM_010510 | IFNβ1 | IFNβ; IFNB | 770 | 175-682 | Mouse IFNβ1 |
| NM_010502 | IFNα1 | IFNα1 | 570 | 53-525 | Mouse IFNα1 |
| NM_008337 | IFNγ | IFN-γ; IFN-gamma | 1207 | 14-573 | Mouse IFNγ |
